# Circulating microRNA profile in humans and mice with congenital GH deficiency

**DOI:** 10.1111/acel.13420

**Published:** 2021-06-12

**Authors:** Tatiana D. Saccon, Augusto Schneider, Cindi G. Marinho, Allancer D. C. Nunes, Sarah Noureddine, Joseph Dhahbi, Yury O. Nunez Lopez, Gage LeMunyan, Roberto Salvatori, Carla R. P. Oliveira, Alécia A. Oliveira‐Santos, Nicolas Musi, Andrzej Bartke, Manuel H. Aguiar‐Oliveira, Michal M. Masternak

**Affiliations:** ^1^ Centro de Desenvolvimento Tecnológico Universidade Federal de Pelotas Pelotas Brazil; ^2^ Burnett School of Biomedical Sciences College of Medicine University of Central Florida Orlando FL USA; ^3^ Faculdade de Nutrição Universidade Federal de Pelotas Pelotas Brazil; ^4^ Division of Endocrinology Health Sciences Graduate Program Federal University of Sergipe Aracaju Brazil; ^5^ Department of Medical Education School of Medicine California University of Science & Medicine San Bernardino CA USA; ^6^ Advent Health Translational Research Institute for Metabolism and Diabetes Orlando FL USA; ^7^ Division of Endocrinology, Diabetes and Metabolism Department of Medicine The Johns Hopkins University School of Medicine Baltimore MD USA; ^8^ Barshop Institute for Longevity and Aging Studies Center for Healthy Aging University of Texas Health Sciences Center at San Antonio and South Texas Veterans Health Care System San Antonio TX USA; ^9^ San Antonio Geriatric Research Education and Clinical Center South Texas Veterans Health Care System San Antonio TX USA; ^10^ Department of Internal Medicine Southern Illinois University School of Medicine Springfield IL USA; ^11^ Department of Head and Neck Surgery Poznan University of Medical Sciences Poznan Poland

**Keywords:** Ames dwarf, dwarfism, IGHD, miRNA

## Abstract

Reduced inflammation, increased insulin sensitivity, and protection against cancer are shared between humans and mice with GH/IGF1 deficiency. Beyond hormone levels, miRNAs are important regulators of metabolic changes associated with healthy aging. We hypothesized that GH deficiency in humans alters the abundance of circulating miRNAs and that a subset of those miRNAs may overlap with those found in GH‐deficient mice. In this study, subjects with untreated congenital isolated GH deficiency (IGHD; *n* = 23) and control subjects matched by age and sex (*n* = 23) were recruited and serum was collected for miRNA sequencing. Serum miRNAs from young (6 month) and old (22 month) Ames dwarf (df/df) mice with GH deficiency and their WT littermates (*n* = 5/age/genotype group) were used for comparison. We observed 14 miRNAs regulated with a genotype by age effect and 19 miRNAs regulated with a genotype effect independent of age in serum of IGHD subjects. These regulated miRNAs are known for targeting pathways associated with longevity such as mTOR, insulin signaling, and FoxO. The aging function was overrepresented in IGHD individuals, mediated by hsa‐miR‐31, hsa‐miR‐146b, hsa‐miR‐30e, hsa‐miR‐100, hsa‐miR‐181b‐2, hsa‐miR‐195, and hsa‐miR‐181b‐1, which target the FoxO and mTOR pathways. Intriguingly, miR‐181b‐5p, miR‐361‐3p, miR‐144‐3p, and miR‐155‐5p were commonly regulated in the serum of humans and GH‐deficient mice. In vitro assays confirmed target genes for the main up‐regulated miRNAs, suggesting miRNAs regulated in IGHD individuals can regulate the expression of age‐related genes. These findings indicate that systemic miRNAs regulated in IGHD individuals target pathways involved in aging in both humans and mice.

## Graphical abstract

In this study we investigated serum regulated miRNAs in GH deficient humans and mice. We observed that some miRNAs are commonly regulated in both species. These miRNAs target pathways involved in aging in both humans and mice.

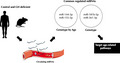

## 1 INTRODUCTION

Growth hormone (GH) is a key hormone regulating longitudinal growth in mammals (Davidson, 1987). Most effects of GH are mediated by insulin‐like growth factor 1 (IGF1) produced mainly in the liver (Jones & Clemmons, 1995). Serum GH levels decrease with age in humans, and it has been suggested this is linked to age‐related health decline (Sonntag et al., 1980). However, the role of GH in aging is complex (Colon et al., 2019; Darcy et al., 2020; Farias Quipildor et al., 2019). Mice with isolated GH deficiency (IGHD) due to ablation of the GHRH gene or mutation of its receptor (Flurkey et al., 2001; Sun et al., 2013), as well as Ames dwarf (df/df) (with combined deficiency of GH, PRL, and TSH) and mice with global disruption of the GH receptor (GHRKO) (Aguiar‐Oliveira & Bartke, 2019; Bartke, 2011), live longer than their WT siblings. Both df/df and GHRKO mice have reduced body size and serum IGF1 levels, increased insulin sensitivity, reduced inflammatory response, and live up to 70% longer than WT littermates (Bartke, 2011).

Reducing insulin‐like signals is associated with extended longevity in several species, including worms, flies, and mice (Tatar et al., 2003). Furthermore, mutations that impair IGF1 signaling are associated with extended longevity in human subjects (Suh et al., 2008; Tazearslan et al., 2011). Much of the research on the role of GH in human longevity has taken advantage of rare experiments in nature. One example is Laron's dwarfism, first described in 1966 in the Mediterranean region (Laron et al., 1966) and later in southern Ecuador, and other parts of the world (Guevara‐Aguirre et al., 1993). This syndrome is characterized by resistance to GH caused by mutations in the GH receptor gene (GHR, OMIM No. 600946). Another example is the dwarfism of Itabaianinha, which we first described in 1999 in the northeasterner Brazilian state of Sergipe (Salvatori et al., 1999), characterized by severe IGHD due to the c.57 + 1G> A mutation in the GHRH receptor gene (*GHRHR*, OMIM No. 612781). Both models have severe dwarfism and visceral obesity, associated with very low or undetectable serum IGF1 concentrations. GH levels are normal or high in Laron dwarfs and very low, but active, in IGHD individuals from the Itabaianinha cohort. Interestingly, an obvious increased longevity was not observed in either of these cohorts, although the highest proportion of deaths is not age‐related (Guevara‐Aguirre et al., 2011). In keeping with findings in mice, GH‐resistant and IGHD humans are partially protected from cancer and diabetes (Guevara‐Aguirre et al., 2011; Laron et al., 2017). Both GH‐deficient mice (Bartke, 2011) and IGHD humans (Oliveira et al., 2012) have high insulin sensitivity with low insulin concentrations. Several studies suggest that high insulin sensitivity is a key factor in GH‐deficient long‐lived individuals (Wijsman et al., 2011). Accordingly, the insulin sensitizer appears to have life‐extending properties that go beyond its glucose‐lowering effect (Bannister et al., 2014). Therefore, understanding how suppression of GH signaling modulates insulin signaling is critical in aging biology.

Recent evidence points to a role for microRNAs (miRNAs) as important regulators of metabolism and healthy aging (Victoria et al., 2017). MicroRNAs are short non‐coding RNA segments that are able to induce target mRNA cleavage and translation repression (Bartel, 2004) and have a central role in post‐transcriptional regulation of cell function (Bartel, 2004). MicroRNAs are also found outside the cell, that is, in the systemic circulation and other body fluids (Cortez et al., 2011; Kolenda et al., 2017; Nunez Lopez et al., 2018). MicroRNAs reach the extracellular environment through passive leakage from damaged cells or active secretion in exosomes (Chen et al., 2012). Circulating miRNAs can target genes in cells from different tissues and organs (Chen et al., 2012). The circulating miRNA signature can potentially serve as non‐invasive diagnosis of chronic diseases such as cancer, diabetes, and cardiovascular disease (Cortez et al., 2011; Vychytilova‐Faltejskova et al., 2016; Zheng et al., 2013). It is also thought that circulating miRNAs can act like endocrine hormones regulating several physiological processes (Chen et al., 2012). For example, we identified a set of circulating miRNAs differentially regulated by aging in GH‐deficient df/df mice (Victoria et al., 2015), further supporting a role for miRNAs in aging modulation. Some of these miRNAs may serve as biomarkers and some may even have a functional role in aging. Based on previous work from our group in mice, we hypothesized that GH deficiency in humans alters the abundance (expression) of circulating miRNAs with known roles in aging/longevity and a subset of those miRNAs will have conserved effects in aging and/or dwarfism from mice to humans.

## 2 RESULTS

### 2.1 Clinical characteristics of IGHD patients

There were equal numbers of women in each group *n* = 11. The number of smokers (IGHD, *n* = 3; WT, *n* = 1), diabetics (IGHD, *n* = 3; WT, *n* = 4), previous diagnosis of arterial hypertension (IGHD, *n* = 8; WT, *n* = 7), and myocardial infarction (IGHD, *n* = 2; WT, *n* = 0) were not different between groups (Table 1). Clinical characteristics are described in Table 1. Overall, IGHD individuals had lower height and weight, but BMI was similar among groups. IGHD individuals also had lower systolic and diastolic blood pressure and creatinine serum concentrations (Table 1).

**TABLE 1:** Clinical characteristics of the studied population

	Control young	IGHD young	Control Old	IGHD Old	*p* Value		
					Group	Age	Age*Group
Number of patients	10	11	13	12			
Age (years)	39.5 (2.5)	39.1 (2.5)	62.6 (2.3)	63.6 (2.6)	0.89	<.0001	0.77
BMI (kg/m^2^)	27.4 (1.8)	22.9 (1.8)	28.4 (1.7)	28.2 (1.7)	0.18	0.08	0.22
Weight (kg)	74.5 (5.5)**^a^**	37.5 (5.5)**^b^**	71.7 (5.0)**^a^**	43.4 (5.2)**^b^**	<.0001	0.77	0.40
Height (m)	1.65 (0.01)**^a^**	1.29 (0.02)**^b^**	1.56 (0.02)**^a^**	1.24 (0.02)**^b^**	<.0001	0.001	0.44
Systolic BP (mm Hg)	121.2 (4.2)**^b^**	100.2 (4.2)**^a^**	135.9 (3.8)**^b^**	132.6 (4.0)**^b^**	0.005	<.0001	0.04
Diastolic BP (mm Hg)	82.5 (2.8)**^a^**	69.3 (2.8)**^b^**	82.7 (2.6)**^a^**	77.4 (2.7)**^b^**	0.001	0.14	0.14
Glucose (mg/dl)	83.6 (11.9)	78.0 (11.9)	113.6 (10.9)	99.6 (11.3)	0.39	0.03	0.71
2‐h glucose (mg/dl)	122.5 (10.5)	106.7 (10.5)	164.0 (11.5)	150.4 (12.2)	0.20	0.0007	0.92
HbA1c	5.4 (0.4)	5.5 (0.4)	6.2 (0.3)	6.4 (0.3)	0.77	0.02	0.91
Cholesterol (mg/dl)	216.4 (13.1)	207.3 (13.1)	211.8 (12.0)	217.5 (12.5)	0.89	0.83	0.56
HDL (mg/dl)	43.6 (2.6)	42.5 (2.6)	44.9 (2.4)	44.0 (2.4)	0.69	0.58	0.97
LDL (mg/dl)	145.1 (11.5)	141.3 (11.5)	141.5 (10.5)	141.9 (10.9)	0.88	0.89	0.85
Triglycerides (mg/dl)	150.8 (21.1)	116.7 (21.1)	127.7 (19.4)	157.8 (21.1)	0.92	0.67	0.12
Creatinine (mg/dl)	0.80 (0.04)**^a^**	0.69 (0.04)**^b^**	0.83 (0.04)**^a^**	0.68 (0.04)**^b^**	0.002	0.82	0.63
Framingham risk score	5.3 (3.3)	3.3 (3.3)	26.5 (3.1)	32.1 (3.2)	0.58	<.0001	0.24

Note

Data are presented as least square means (SEM). Different superscript letters indicate statistical difference at *p* < 0.05.

### 2.2 Circulating miRNAs in IGHD humans and mice

**FIGURE 1:**
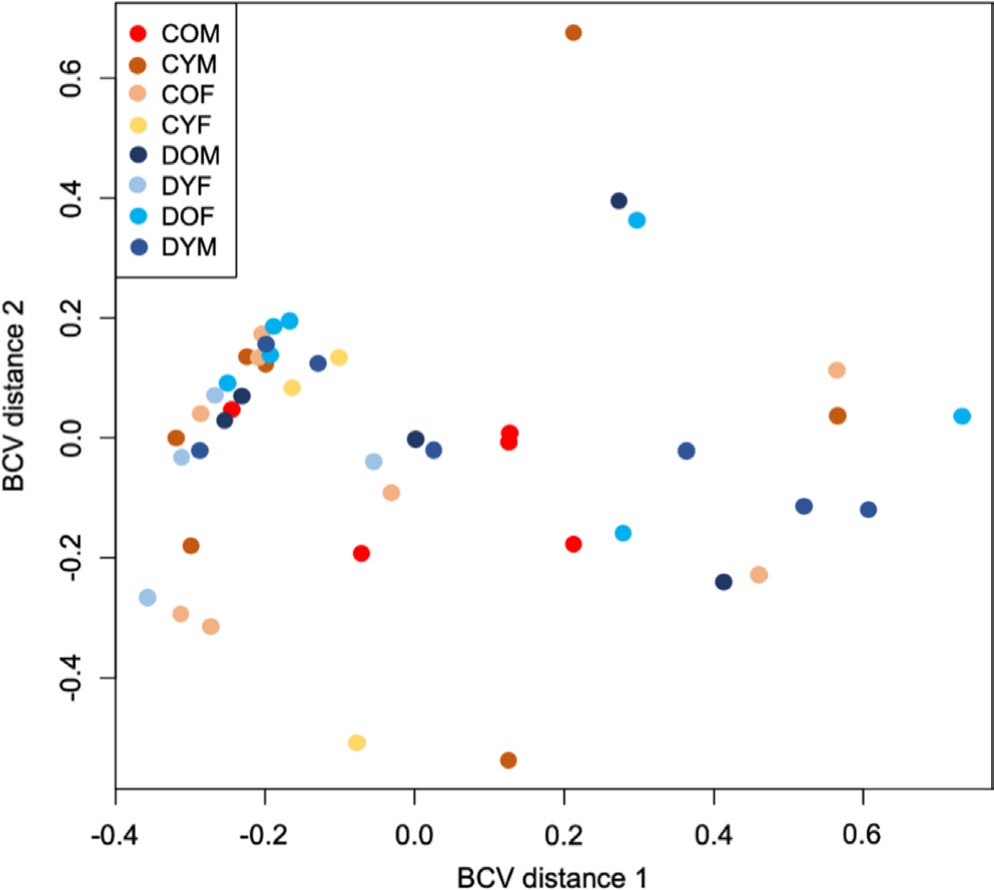
Principal components analysis of serum miRNAs in untreated isolated congenital GH deficiency (IGHD) patients. Samples were grouped by genotype (Control vs IGHD), sex, and age (young, 32–45 years; and old, 45–82 years). Control old males (COM), control young males (CYM), control old females (COF), control young females (CYF), IGHD old males (DOM), IGHD young males (DYM), IGHD old females (DOF), IGHD young females (DYF)

After sequencing and processing the RNA‐Seq data from serum samples, an average of 31,262,811 ± 9,837,722 filtered reads per sample was obtained with an 8.3 ± 0.5% overall alignment rate to mature miRNAs in the human genome. In total, 437 known miRNAs were identified in the serum samples. PCA analysis of the samples indicates significant overlap among genotype and age groups (Figure 1).

In mice, an average of 12,099,035 ± 2,716,859 filtered reads per sample was obtained with a 20.3 ± 3.1% overall alignment rate to mature miRNAs. Overall, 298 known miRNAs were identified in serum from mice.

### 2.3 Circulating miRNAs correlation with clinical characteristics

**FIGURE 2:**
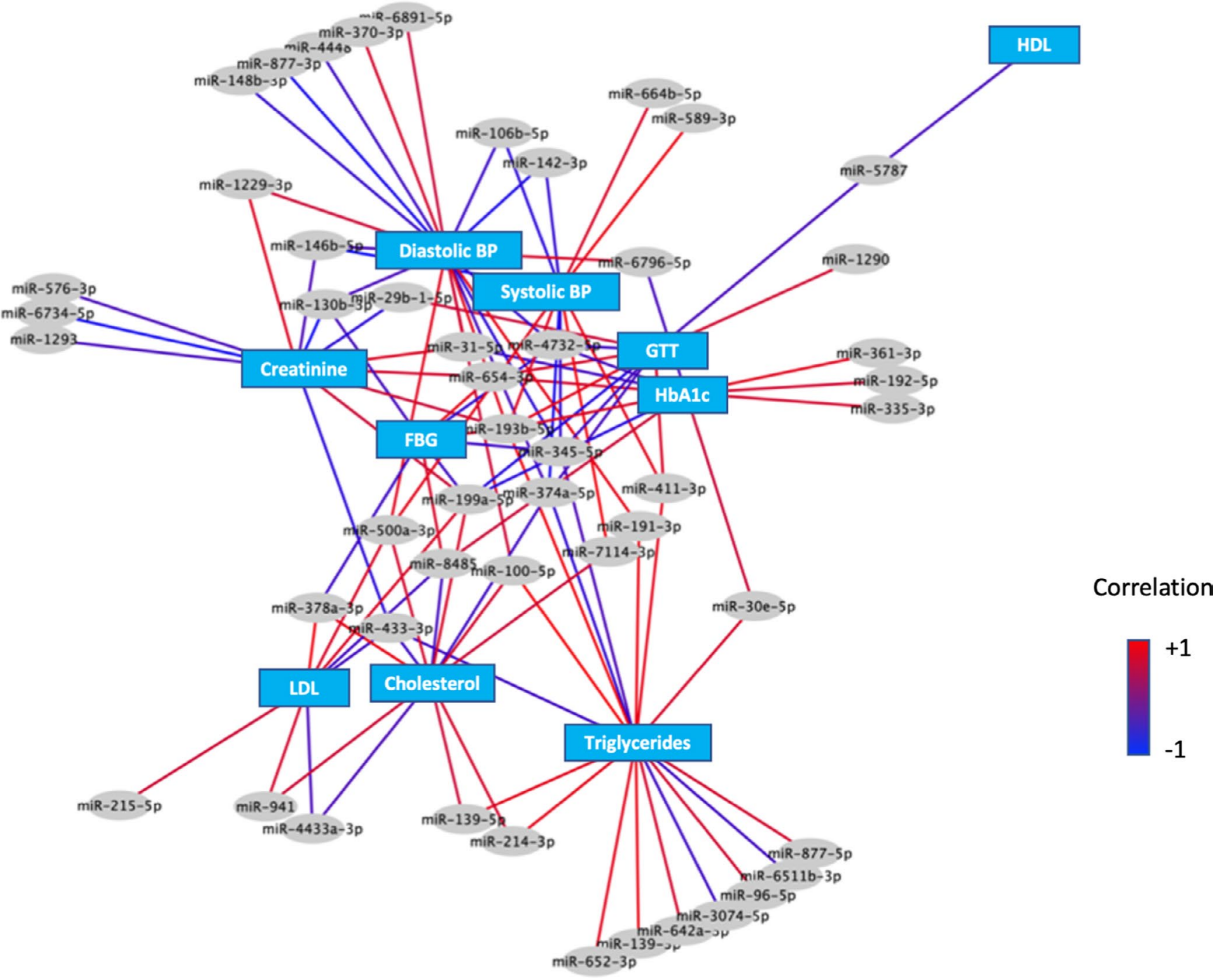
Regulatory networks of serum miRNAs and clinical parameters in young and old untreated isolated congenital GH deficiency (IGHD) patients. Blue boxes indicate clinical parameters and the gray ellipses serum miRNAs. The connection lines indicate significant correlation between clinical parameters and miRNAs

The correlation of clinical parameters with miRNA expression level is presented in Table S1. In Figure 2, we presented a network that highlighted the significant pairwise correlations between miRNA levels and clinical measures. Interestingly, hsa‐miR‐193b‐5p, hsa‐miR‐199a‐5p, hsa‐miR‐654‐3p, and hsa‐miR‐4732‐5p were simultaneously correlated to fasting glucose, 2‐h GTT, and HbA1c, indicating their association to glucose metabolism.

### 2.4 Circulating miRNAs displaying significant IGHD genotype by age effect in humans and mice

Table 2 presents the list of regulated miRNAs with a genotype by age interaction in the serum of IGHD and control subjects. We identified 14 miRNAs with a genotype by age interaction. In Figure S1, these miRNAs are represented as box plots. Considering all genotype by age‐regulated miRNAs in GH‐deficient df/df mice (Table S1), we observed that miR‐144‐3p and miR‐155‐5p were commonly regulated with human genotype by age miRNAs.

**TABLE 2:** List of regulated microRNAs (miRNAs) with a genotype by age interaction and genotype effect in the serum of IGHD and control subjects

miRNAs	Genotype by age		Genotype effect		Genotype effect		Genotype effect	
	Interaction		IGHD vs. Control		Young only		Old only	
	LogFC^a^	*p* Value	LogFC^a^	*p* Value	LogFC^a^	*p* Value	LogFC^a^	*p* Value
miRNAs with a genotype by age effect								
hsa‐miR‐15a‐5p	**−5.4**	**0.00002**	0.2	0.84498	**2.8**	**0.00233**	**−2.6**	**0.00167**
hsa‐miR‐144‐3p	**−3.3**	**0.00840**	−0.7	0.51586	1.3	0.13723	**−2.0**	**0.01573**
hsa‐miR‐6721‐5p	**2.0**	**0.01613**	−0.6	0.44111	**−1.3**	**0.03247**	0.7	0.21055
hsa‐miR‐411‐3p	**2.1**	**0.02544**	0.5	0.62706	−0.8	0.24715	**1.3**	**0.04139**
hsa‐miR‐4787‐5p	**2.2**	**0.02780**	−1.0	0.34911	**−1.6**	**0.04686**	0.6	0.37536
hsa‐miR‐155‐5p	**−3.5**	**0.03043**	0.2	0.88465	1.9	0.11944	−1.6	0.12815
hsa‐miR‐345‐5p	**−3.1**	**0.03271**	2.0	0.16733	**2.5**	**0.01807**	−0.6	0.55159
hsa‐miR‐193b‐5p	**3.1**	**0.03546**	−1.4	0.30134	**−2.3**	**0.03080**	0.8	0.39379
hsa‐miR‐543	**3.0**	**0.03686**	−1.8	0.20780	**−2.4**	**0.02558**	0.6	0.52782
hsa‐miR‐6880‐5p	**2.4**	**0.04018**	−1.1	0.38192	−1.8	0.05585	0.7	0.38657
hsa‐miR‐1290	**2.8**	**0.04341**	−0.1	0.93288	−1.5	0.16032	1.4	0.14584
hsa‐miR‐576‐3p	**1.8**	**0.04692**	0.3	0.72960	−0.7	0.21021	1.0	0.08710
hsa‐miR‐6511b‐3p	**−2.7**	**0.04839**	**−2.6**	**0.04589**	0.0	0.97692	**−2.6**	**0.00420**
hsa‐miR‐191‐3p	**2.8**	**0.04970**	0.1	0.92235	−1.3	0.20647	1.5	0.12243
miRNAs with a genotype effect								
hsa‐miR‐1249‐3p	−1.3	0.25044	**−3.7**	**0.00083**	−1.2	0.12864	**−2.5**	**0.00134**
hsa‐miR‐146b‐5p	−0.6	0.66377	**4.6**	**0.00088**	**2.6**	**0.01026**	**2.0**	**0.02051**
hsa‐miR‐4433a‐5p	0.9	0.40643	**−3.6**	**0.00303**	**−2.3**	**0.01090**	−1.4	0.07083
hsa‐miR‐4485‐3p	0.1	0.96448	**3.2**	**0.00516**	1.6	0.06059	**1.6**	**0.04696**
hsa‐miR‐361‐3p	−1.0	0.46619	**3.8**	**0.00686**	**2.4**	**0.02067**	1.4	0.12174
hsa‐miR‐4433b‐3p	0.8	0.59789	**−3.9**	**0.00840**	**−2.3**	**0.03231**	−1.6	0.10221
hsa‐miR‐1229‐3p	−0.3	0.84025	**−3.9**	**0.01162**	−1.8	0.10725	**−2.1**	**0.03093**
hsa‐miR‐195‐5p	−0.2	0.86620	**2.9**	**0.01232**	1.5	0.06904	1.3	0.09913
hsa‐miR‐6891‐5p	0.4	0.64629	**−2.1**	**0.01268**	**−1.3**	**0.04586**	−0.9	0.11003
hsa‐miR‐181b‐5p	0.8	0.51927	**2.9**	**0.02005**	1.1	0.24452	**1.9**	**0.02628**
hsa‐miR‐31‐5p	−0.1	0.94594	**−2.8**	**0.02596**	−1.4	0.13790	−1.4	0.06912
hsa‐miR‐4448	−1.0	0.34270	**2.4**	**0.02611**	**1.7**	**0.03255**	0.7	0.33965
hsa‐miR‐192‐5p	−1.2	0.39722	**3.1**	**0.02843**	**2.2**	**0.04149**	1.0	0.29402
hsa‐miR‐6803‐3p	−0.9	0.38390	**−2.2**	**0.03401**	−0.7	0.38527	**−1.6**	**0.02585**
hsa‐miR‐30e‐5p	1.1	0.33377	**2.3**	**0.03870**	0.6	0.45658	**1.7**	**0.02703**
hsa‐miR‐100‐5p	1.5	0.18070	**2.3**	**0.03947**	0.4	0.61292	**1.9**	**0.01209**
hsa‐miR‐106b‐5p	0.9	0.51597	**3.1**	**0.04335**	1.1	0.33553	**2.0**	**0.04098**
hsa‐miR‐130b‐3p	0.2	0.85880	**2.7**	**0.04509**	1.2	0.21244	1.5	0.09912
hsa‐miR‐5787	−1.5	0.15113	**2.2**	**0.04890**	**1.8**	**0.02512**	0.3	0.62108

^a^ LogFC: difference of the mean of logarithm of the of fold change of the compared groups.

LogFC and *P* values marked in bold represent miRNAs with significant difference.

### 2.5 Circulating miRNAs displaying significant IGHD genotype effect in humans and mice

Table 2 shows the list of regulated miRNAs with a genotype effect in the serum of IGHD and control subjects. We identified 19 miRNAs regulated by genotype independent of age. In Figure S2, these miRNAs are represented as box plots. When considering genotype‐regulated miRNAs in GH‐deficient df/df mice (Table S2), we observed that miR‐181b‐5p and miR‐361‐3p were up‐regulated in both mice and humans with GH deficiency. However, a third common miRNA, miR‐192‐5p, was down‐regulated in mice and up‐regulated in humans with GH deficiency. Considering miRNA families, we observed that mir‐15, mir‐361, mir‐146, mir‐192, mir‐181, and mir‐30 families were commonly regulated by genotype in mice and humans.

### 2.6 Interaction network of validated miRNA–mRNA targeting events in IGHD humans

**FIGURE 3:**
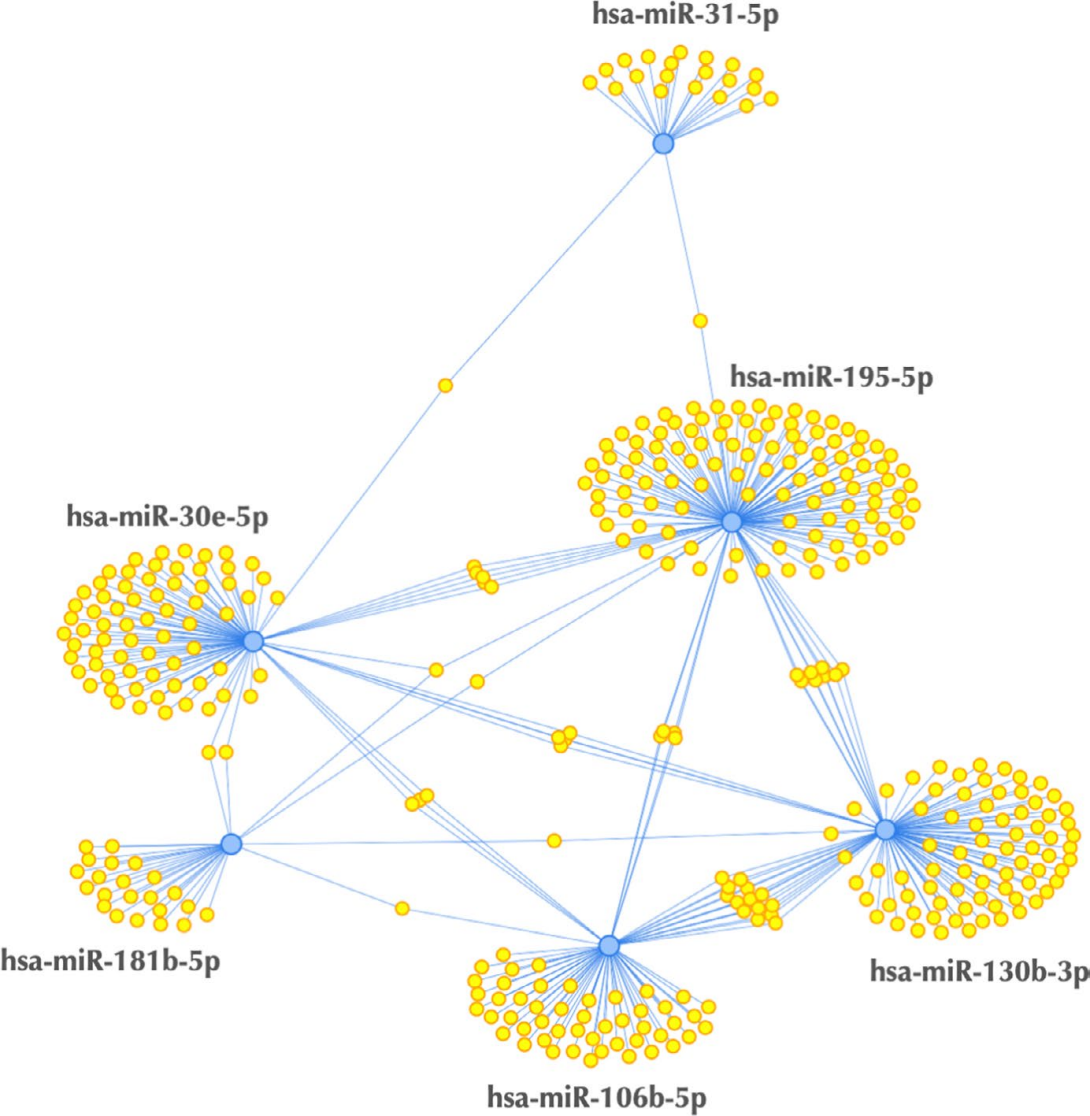
Regulatory networks of miRNA‐over targeted transcripts enriched for miR‐31‐5p, miR‐195‐5p, miR‐30e‐5p, miR‐130b‐3p, miR‐106b‐5p, and miR‐181b‐5p. Blue circles indicate miRNAs and yellow circles indicate predicted target genes. This figure was generated using the MIENTURNET web tool

The analysis of miRNA–mRNA networks identified a network composed of miR‐31‐5p, miR‐195‐5p, miR‐30e‐5p, miR‐130b‐3p, miR‐106b‐5p, and miR‐181b‐5p and its target genes as functionally enriched, highlighting the association of this set of miRNAs with aging (Figure 3). The main KEGG pathways regulated by target genes of the regulated miRNAs are shown in Table 3 (complete set in Table S3) for miRNAs with a genotype by age effect and in Table 4 (complete set on Table S4) for miRNAs with an overall genotype effect. Interestingly, these included classically longevity‐associated pathways such as FoxO, mTOR, and insulin signaling pathways. The gene targets of regulated miRNAs for these pathways are shown in Figures S3–S5. Additionally, Table 5 shows a list of overrepresented functions targeted by miRNAs regulated by genotype in humans. No significant functions were affected by genotype by age‐regulated miRNAs. Notably, aging was the top affected function by genotype‐regulated miRNAs and the miRNAs involved were also found in the functionally enriched network from Figure 2. Moreover, insulin resistance and inflammation were also identified as regulated functions by genotype‐regulated miRNAs.

**TABLE 3:** Pathways regulated by target genes of miRNAs regulated by the genotype by age interaction

KEGG pathway	*p*‐Value	Genes	miRNAs
Aging‐related pathways			
MAPK signaling pathway	0.002	34% (99/294)	12
PI3K‐Akt signaling pathway	0.015	34% (119/354)	12
FoxO signaling pathway	3.21E‐04	47% (61/131)	12
Prolactin signaling pathway	3.21E‐04	50% (35/70)	12
mTOR signaling pathway	0.001	21% (32/155)	11
Type II diabetes mellitus	0.002	54% (25/46)	11
Insulin signaling pathway	0.004	43% (59/137)	11
TGF‐beta signaling pathway	0.001	39% (37/94)	10
Cancer‐related pathways			
Pathways in cancer	0.006	26% (137/531)	14
Choline metabolism in cancer	0.001	48% (47/98)	13
Proteoglycans in cancer	1.37E‐05	38% (77/205)	13
Acute myeloid leukemia	0.013	39% (26/67)	13
Glioma	1.18E‐05	43% (32/75)	13
Transcriptional misregulation in cancer	0.008	33% (64/192)	12
Prostate cancer	1.10E‐04	46% (45/97)	12
Melanoma	0.004	46% (33/72)	12
Renal cell carcinoma	0.001	48% (33/69)	11
Endometrial cancer	0.002	45% (26/58)	11
Colorectal cancer	0.003	33% (28/86)	11
Non‐small cell lung cancer	3.21E‐04	40% (29/72)	11
Pancreatic cancer	0.008	39% (30/76)	10

**TABLE 4:** Pathways regulated by target genes of miRNAs regulated by the genotype

KEGG pathways	*p*‐Value	Genes	miRNAs
Up‐regulated miRNAs			
Aging‐related pathways			
TGF‐beta signaling pathway	1.74E‐09	38% (36/94)	8
FoxO signaling pathway	3.52E‐05	42% (55/131)	8
mTOR signaling pathway	3.26E‐04	19% (30/155)	9
AMPK signaling pathway	3.79E‐04	43% (51/120)	10
Prolactin signaling pathway	0.007	40% (28/70)	7
Insulin signaling pathway	0.010	38% (52/137)	11
Cancer‐related pathways			
Glioma	2.97E‐08	44% (33/75)	8
Proteoglycans in cancer	1.03E‐07	36% (74/205)	11
Prostate cancer	2.16E‐05	44% (43/97)	9
Acute myeloid leukemia	7.05E‐05	43% (29/67)	9
Pathways in cancer	4.10E‐04	25% (132/531)	12
Melanoma	0.002	43% (31/72)	7
Pancreatic cancer	0.005	38% (29/76)	8
Down‐regulated miRNAs			
Biotin metabolism	3.41E‐06	33% (1/3)	1
Lysine degradation	0.038	10% (6/63)	4
Thyroid hormone signaling pathway	0.040	10% (12/121)	5

**TABLE 5:** Overrepresented function in genotype‐regulated miRNAs

Term	Count	%	Fold	*p*‐Value	FDR	miRNA
Aging	7	11	7.1	<0.0001	0.014	hsa‐mir‐31, hsa‐mir‐146b, hsa‐mir‐30e, hsa‐mir‐100, hsa‐mir‐181b‐2, hsa‐mir‐195, hsa‐mir‐181b‐1
Peritoneal cavity homeostasis	4	17	11.1	<0.0001	0.043	hsa‐mir‐31, hsa‐mir‐146b, hsa‐mir‐100, hsa‐mir‐192
Inflammation	7	6	4.0	0.001	0.077	hsa‐mir‐146b, hsa‐mir‐100, hsa‐mir‐181b‐1, hsa‐mir‐192, hsa‐mir‐130b, hsa‐mir‐31, hsa‐mir‐181b‐2
Insulin resistance	3	17	10.6	0.002	0.096	hsa‐mir‐195, hsa‐mir‐181b‐1, hsa‐mir‐181b‐2

Abbreviation: FDR, false discovery rate.

### 2.7 In vitro validation of target genes of serum‐regulated miRNA in IGHD humans

Based on the up‐regulation of miR‐100‐5p, miR‐195‐5p, miR‐181b‐5p, and miR‐30e‐5p in serum of IGHD patients and their association to age‐related target genes, we transfected these four miRNAs (individually and as a mix) *in vitro*. We observed significant up‐regulation of these miRNAs in cells in culture, establishing the effectiveness of the transfection (Figure S6). In the individual miRNA transfection, we observed the down‐regulation of mTOR by miR‐100‐5p mimic (Figure S7), AKT and IRS1 tended to be down‐regulated by miR‐195 and miR‐30e, respectively, while there was no visible impact of miR‐181b on its target, NFκβ (Figure S7). Importantly, in the combined transfection mix, we observed significant down‐regulation of the age‐related genes mTOR, AKT, NFκβ, and IRS‐1 (Figure S8). Other non‐targeted genes were also tested and were not affected by the miRNA transfection mix (Figure S8).

## 3 DISCUSSION

In the current study, we identified circulating serum miRNAs regulated between IGHD subjects and age/gender‐matched controls. The only clinical differences between the two groups were the lower blood pressure in IGHD subjects (although normal in both groups) and reduced creatinine serum concentrations, reflecting lower muscular mass in IGHD subjects (Aguiar‐Oliveira & Bartke, 2019). These sera‐regulated miRNAs target pathways known for their role on longevity, including mTOR, Insulin, and FoxO. Accordingly, aging was identified as the top overrepresented function regulated by this set of miRNAs. In addition, we observed that some of the serum‐regulated miRNAs in human subjects overlap with miRNAs regulated in serum of GH‐deficient df/df mice, pointing toward some translational potential of these miRNAs. Importantly, this overlapping between dwarfing syndromes in humans and mice further supports the use of the Ames dwarf mice as a model to study aging in humans. In vitro assays confirmed target genes for the main up‐regulated miRNAs. We highlight that hsa‐miR‐31, hsa‐miR‐146b, hsa‐miR‐30e, hsa‐miR‐100, hsa‐miR‐181b‐2, hsa‐miR‐195, and hsa‐miR‐181b‐1 were overrepresented for the aging function and for targeting the FoxO and mTOR pathways, and therefore, we focused the discussion on these miRNAs. Based also on the overlap with miRNAs regulated in GH‐deficient df/df mice, we considered hsa‐miR‐15a‐5p, hsa‐miR‐144‐3p, has‐miR‐155‐5p, and hsa‐miR‐361‐3p interesting targets for further discussion.

A previous study observed that miR‐106b and miR‐130b were down‐regulated with age in human serum (Zhang et al., 2015). Here, we find that these miRNAs were up‐regulated in IGHD subjects, suggesting prevention of the decline of these miRNAs in IGHD individuals. In addition, miR‐361 and miR‐192 were commonly up‐regulated in our study and in a previous study evaluating the miRNA profile from B cells of centenarians (Gombar et al., 2012). The miR‐361 was also up‐regulated in the long‐living GH‐deficient Ames dwarf mice, suggesting conservation of this miRNA. Gombar et al. also observed that miR‐181a and miR‐181c were up‐regulated in centenarians. We observed that miR‐181b, from the same family, was up‐regulated in IGHD individuals and in GH‐deficient Ames dwarf mice. Another study using mononuclear cells from centenarians observed significant up‐regulation of miR‐130a compared to octogenarians (Serna et al., 2012). We observed that miR‐130b, from the same family, was up‐regulated in IGHD individuals. In the Baltimore longitudinal study of aging, miR‐130 was among the top up‐regulated miRNA in serum of long‐lived individuals (Smith‐Vikos et al., 2016). We observed that miR‐130 was predicted to be involved in the regulation of inflammation and significantly involved in the network of regulated genes and miRNAs along with miR‐181. The miR‐130 was also predicted to target the FoXO, insulin, and mTOR pathways. It also targets IGF1 (Li et al., 2016), which may indicate a common link between the GH‐deficient humans and mice and long‐lived individuals and centenarians. This is consistent with research indicating that mutations in IGF1R gene in humans are also associated with longevity (Suh et al., 2008).

We observed that serum miRNAs were significantly correlated to several clinical parameters measured in this population. A total of 50 miRNAs had some degree of correlation with clinical parameters, of which 33 miRNAs showed differential genotype or genotype by age regulation between IGHD and control individuals. Although this evidence is only correlative, it suggests that some of these miRNAs may be involved in the pathogenesis of conditions associated with IGHD. Notably, we observed that hsa‐miR‐193b‐5p, hsa‐miR‐199a‐5p, hsa‐miR‐654‐3p, and hsa‐miR‐4732‐5p levels correlated with several glucose metabolism parameters in this population. Serum miR‐193b is known to be involved in the pathogenesis of diabetes and is increased in serum of glucose‐intolerant mice and prediabetic humans (Párrizas et al., 2015). The miR‐199a is also increased in serum of patients with type 2 diabetes mellitus (T2DM) and inhibits GLUT4 expression *in vitro* (Yan et al., 2014). Serum miR‐4732 has also been linked with diabetes in humans (Wander et al., 2020). As previously mentioned, IGHD individuals have high insulin sensitivity with low insulin concentrations (Oliveira et al., 2012). Therefore, future studies should focus on this specific set of miRNAs and its potential impact on the regulation of glucose metabolism and longevity.

We found that miR‐181b‐5p was up‐regulated by approximately sevenfold in IGHD humans and the effect was even more pronounced in older individuals; the same pattern was observed in GH‐deficient df/df mice. This miRNA was identified as regulating the aging function as well as the mTOR pathway, emerging as an interesting target for aging studies. Previous observations showed that miR‐181b‐5p decreases with age in mice, with a fourfold reduction from three to eight months of age and eightfold reduction from eight to 12 months of age (Kim et al., 2017). This suggests that the reduction of miR‐181 normally observed with aging is not occurring in IGHD humans or in long‐living df/df mice. Similar to the effect of aging, a chronic high‐fat diet reduced miR‐181b serum levels in mice (Copier et al., 2017). The miR‐181b inhibits the NF‐κB signaling pathway and consequently has anti‐inflammatory effects (Sun et al., 2014). This fits with observations that inflammation increases with age; however, this is prevented in GH‐deficient mice (Masternak & Bartke, 2012). In fact, in the current study we observed that inflammation was one of the regulated functions, including miR‐181b. Thus, maintenance of high levels of miR‐181b in IGHD individuals may mitigate age‐related inflammatory and degenerative conditions, including insulin resistance. Consistently with the effects of aging, miR‐181b‐5p levels were significantly decreased in serum and liver of diabetic mice as well as the serum of T2DM patients (Wang et al., 2019). Hepatic inhibition of miR‐181b‐5p led to insulin resistance in mice (Wang et al., 2019). Conversely, miR‐181b expression increased in human senescent keratinocytes, suggesting tissue‐specific proprieties of this miRNA (Rivetti di Val Cervo et al., 2012). The miR‐181b overexpression also inhibited cell proliferation, migration, invasion, and tumorigenesis by targeting IGF‐1R and its downstream signaling pathways (Shi et al., 2013). Therefore, besides preventing inflammation and diabetes, miR‐181 has a tumor suppressor effect, all key factors in age‐related mortality. These are ameliorated in GH‐resistant individuals (Guevara‐Aguirre et al., 2011) and in IGHD individuals from Itabaianinha (Oliveira et al., 2012). The miR‐181b was included in the cell transfection mix and we observed an associated reduction in NFκB and mTOR gene expression, which were predicted targets for this miRNA.

The regulation pattern for serum miR‐30e was like that observed for miR‐181b. IGHD individuals maintained high levels of miR‐30e, while control individuals had a decline in its levels with age, leading to a greater difference in older individuals. A previous study has shown that miR‐30e‐5p decreases with age in human blood (Lai et al., 2014), in agreement with our findings in the control subjects. Similar to miR‐181b, miR‐30e levels were reduced in serum of mice with obesity induced by a high‐fat diet (Copier et al., 2017), suggesting an association with the insulin‐resistant‐associated obesity phenotype. Plasma miR‐30e‐5p level is also reduced in type 1 diabetic patients (Dieter et al., 2019) and is associated with better recovery from cardiac events (Marfella et al., 2013), which overall reinforces its association to metabolic health. In addition, miR30e‐5p is down‐regulated in tumor tissues of breast cancer (BC) patients (Liu et al., 2017). The overexpression of miR‐30e inhibited cell proliferation by targeting IRS1 and blocking the activation of AKT and ERK1/2 pathways (Liu et al., 2017). Our in vitro study confirmed that inclusion of miR‐30e in the transfection mix was associated with reduced gene expression for IRS‐1 and AKT. Therefore, similarly to miR‐181b, miR‐30e serum levels can influence insulin resistance and carcinogenesis, key age‐related conditions.

Another age‐related miRNA that was up‐regulated in IGHD individuals was miR‐100‐5p, with a more prominent effect in older individuals. This miRNA was not regulated in serum of GH‐deficient mice. The miRNA‐100 was shown to inhibit carcinogenesis by directly targeting mTOR (Xu et al., 2013), a finding confirmed by other studies (Ye et al., 2015). We also confirmed reduced mTOR expression in vitro with the addition of miR‐100 individually and in the transfection mix. mTOR is a critical pathway to regulate aging in different species (Johnson et al., 2013). Several interventions that successfully slow down aging target the mTOR pathway (Johnson et al., 2013). In the whole IGHD Itabaianinha cohort, during 25 years of medical care, our team did not diagnose any cases of breast, colon, or prostate cancers (Aguiar‐Oliveira & Salvatori, 2020), suggesting reduced risk of cancer, as shown in Ecuadorian GH‐resistant individuals (Guevara‐Aguirre et al., ,^1993^, ^2011^). On the other hand, these individuals exhibit a low, but not negligible, frequency of diabetes (15.6%, assessed by an oral glucose tolerance test) (Vicente et al., 2013), while there was no self‐reported diagnosis of diabetes in the Ecuadorian GH‐resistant cohort.

Expression of miR‐100 in visceral adipose tissue was decreased in obese subjects with T2DM (Pek et al., 2016). The miR‐100 was shown to be down‐regulated in blood of type 2 (He et al., 2017) and type 1 diabetic patients (He et al., 2017). Indeed, the insulin signaling pathway was regulated by both genotype by age and genotype in IGHD patients. Although miR‐100‐5p was regulated in IGHD individuals, we did not observe its regulation in mice. Similarly, miR‐100‐5p is a serum marker of diabetes type 1 in humans but not in mice (Assmann et al., 2017). Regarding the inflammatory profile, miR‐100 overexpression shifted macrophages toward M2 polarization (Wang et al., 2018), suggesting a less pro‐inflammatory profile. This is also supported by our observation of reduced NFκB gene expression in vitro for cells treated with the miRNA mix. Available evidence also suggests that miR‐100 is down‐regulated in many human cancers including breast, bladder and lung, by regulating proliferation, differentiation, migration, invasion, and apoptosis due to targeting the mTOR pathway (Li et al., 2015). Therefore, miR‐100‐5p is a potential target for age‐related interventions as it focuses on key aging pathways and is strongly up‐regulated in IGHD individuals.

miR‐195‐5p was also identified as an important miRNA, as it was up‐regulated in IGHD individuals. In mice, we observed that mmu‐miR‐195a‐5p, which has an identical sequence to hsa‐miR‐195‐5p, had a significant genotype by age effect. The miR‐195‐5p levels were decreased in serum of humans and mice with acute kidney injury (Xu et al., 2020). Injection of a miR‐195‐5p mimic in mice reduced oxidative stress and pro‐inflammatory cytokine production after kidney injury (Xu et al., 2020). In addition, miR‐195‐5p expression was down‐regulated in tissue and cell lines from lung cancer (Wang et al., 2014). The miR‐195‐5p can inhibit IGF‐1R and has a tumor‐suppressive effect (Wang et al., 2014). Conversely, miR‐195‐5p directly binds to Sirt1 3′ UTR, inhibiting its expression (Kondo et al., 2016). This is unexpected as SIRT1 is an important mediator of several beneficial effects of caloric restriction in mice (Canto & Auwerx, 2009). Overall, this miRNA targets inflammation and carcinogenesis, pivotal for healthy aging. The miR‐195‐5p was also included in our transfection mix in vitro and was associated with reduced expression of AKT, as predicted. The miR‐361‐3p was also up‐regulated in serum of IGHD individuals and mice in our study. The miR‐361‐3p is another important cancer biomarker, with down‐regulation in serum of lung cancer patients (Roth et al., 2012). Furthermore, higher levels of miR‐361‐3p were associated with improved survival after diagnosis of cervical cancer (Liu et al., 2018). Decreased miR‐361‐3p promoted cell growth, proliferation, invasion, and migration in vitro, and proliferation and metastasis in vivo (Chen et al., 2016). Therefore, like other miRNAs previously discussed, up‐regulation of miR‐361‐3p can reduce cancer growth and can be associated with lower cancer incidence in IGHD individuals.

One of the genotypes by age‐regulated miRNAs was miR‐15a‐5p, which belongs to the miR‐15 superfamily along with miR‐195‐5p. We observed an interesting pattern for miR‐15a levels, as it was increased in serum of young IGHD individuals, while decreased in serum of older IGHD subjects. We also observed that miR‐15a was reduced in serum of older GH‐deficient df/df mice, although no changes were observed in younger mice. Over‐expression of miR‐15a/b in porcine pre‐adipocytes inhibited FoxO1 expression and promoted adipocyte differentiation and lipid accumulation (Dong et al., 2014). Additionally, miR‐15a overexpression promoted insulin biosynthesis in mouse islet cells, whereas its repression inhibited insulin biosynthesis (Sun et al., 2011). The miR‐15a regulated insulin biosynthesis by targeting uncoupling protein‐2 (UCP‐2) gene expression (Sun et al., 2011). The miR‐15a down‐regulation was also observed in several tumors (Finnerty et al., 2010) and serum (Li et al., 2017). The reduction of serum miR‐15a in older individuals may have a role in conferring cancer protection for IGHD subjects (Aguiar‐Oliveira & Bartke, 2019). Similarly, serum levels of miR‐15a were strongly up‐regulated in acute ischemic stroke patients (Wu et al., 2015). Two other important miRNAs with significant genotype by age regulation were miR‐144‐3p and miR‐155‐5p. We observed that miR‐144‐3p was down‐regulated in older IGHD human subjects. The same was observed in Ames dwarf mice, suggesting a conserved mechanism for this miRNA. Importantly, miR‐144 down‐regulated IRS1 mRNA and protein levels and correlated with glucose levels in mice (Karolina et al., 2011). Additionally, miR‐144 was highly up‐regulated in T2DM patients (Gallagher et al., 2010; Karolina et al., 2011). Therefore, the miR‐144 down‐regulation in IGHD humans seen here is in line with the finding that this population is more insulin sensitive (Oliveira et al., 2012), despite exhibiting visceral obesity (Gomes‐Santos et al., 2014). Caloric restriction in rodents is known to elicit several life‐extending physiological benefits similar to GH deficiency. As with GH deficiency, caloric restriction decreased miR‐144 expression in rats (Csiszar et al., 2014). Furthermore, miR‐144 up‐regulation was associated with decreased expression of NRF2, an important regulator of the oxidative stress response, and decreased miR‐144 expression induced by caloric restriction rescued NRF2 expression and reduced oxidative stress (Csiszar et al., 2014). This provides further evidence for the role of miR‐144 in aging‐related pathologies. In addition, miR‐144‐3p overexpression accelerated lipid accumulation in adipocytes and positively regulated adipogenesis (Shen et al., 2018). Intravenous injection of miR‐144‐3p promoted adipogenesis in mice (Shen et al., 2018), providing further evidence for a possible functional role of decreased serum miR‐144 in IGHD individuals. Moreover, miR‐144‐3p mimics increased expression of inflammatory factors, IL‐1b, IL‐6, and TNF‐a, in vivo and in vitro (Hu et al., 2014). Current literature points to a role of miR‐144 in insulin sensitivity, oxidative stress, inflammation, and adipogenesis, all of which are hallmarks of aging. Further functional studies in animals with slower or accelerated aging are needed to confirm these findings. Regarding miR‐155‐5p, higher levels were observed for young IGHD individuals. In young individuals, the higher levels of miR‐155‐5p may be beneficial due to its contribution to a faster response to infection or injury (Faraoni et al., 2009). On the other hand, as age advances and humans are exposed and accumulate cellular damage and cancer risk (Belpomme et al., 2007; White et al., 2014), the lower miR‐155‐5p levels in old IGHD patients may contribute to decreased inflammation‐related cancer risk in this population (Tili et al., 2011). Moreover, reduced miR‐155‐5p in older individuals can contribute to a reduced inflammatory milieu (Olivieri et al., 2013) that likely contributes to improved organismal homeostasis and fewer chronic conditions.

Besides the classical aging‐associated pathways (FoXO, mTOR and insulin), we observed that the prolactin (PRL) signaling pathway was targeted by miRNAs up‐regulated in IGHD subjects. Prolactin signaling pathway was also targeted by genotype by age‐regulated miRNAs. Previous evidence indicates that PRL levels are higher in older rats (Esquifino et al., 2004). Similar observations were made in humans, although the increase in PRL with age was more obvious in men than women (Sawin et al., 1989). Among the target genes in the PRL pathway, we observed Pi3k/Akt/FoXO, which are genes involved in aging (Kenyon, 2010). In this context, it is important to mention that Ames dwarf mice are also deficient in PRL, besides GH and TSH (Slabaugh et al., 1981). Despite these observations, studies pointed out that PRL knockout mice have negligible metabolic alterations (LaPensee et al., 2006). Growth hormone and PRL pathways share the intracellular Jak/STAT signaling (Goffin & Kelly, 1997). We observed that these up‐regulated miRNAs targeted STAT3. Recent evidence suggests the JAK/STAT pathway is more active in senescent cells and that its inhibition can alleviate age‐related tissue dysfunction (Xu et al., 2016).

While congenital IGHD in the Itabaianinha cohort has many similarities with the GH‐resistance syndrome of the Laron dwarfs, some differences exist. Growth hormone‐resistant infants in both Israeli and Ecuadorian cohorts exhibit microphallus, and most of them have reduced penis and testes size in adulthood, despite normal blood androgens and reproductive potential (Laron & Kauli, 2016). However, microphallus is absent in the Itabaianinha cohort. This suggests that having minimal GH secretion can have a growth effect on the external genitalia directly or via local IGF1. There seems to be a discrepancy between the Ecuadorian and the Israeli cohorts of Laron dwarfs in terms of cognitive function, reported to be normal or improved in the former (Nashiro et al., 2017), and somehow compromised in the latter (Laron, 2011). Conversely, the cerebral vascular function is normal in the Itabaianinha subjects (Marinho et al., 2020), and they seem to have no obvious cognitive problems. There are also some discrepancies in the progression of the atherosclerosis in Israeli and Ecuadorian cohorts of Laron patients, apparently slower in the latter. The progression of atherosclerosis is not premature in Itabaianinha (Costa et al., 2016), and they have normal longevity (Aguiar‐Oliveira et al., 2010). The maximal individual lifespan was 85 years in the Ecuadorian Laron patients (Nashiro et al., 2017; Rosenfeld et al., 1994; Scheinowitz et al., 2011) and 103 years in the Itabaianinha cohort (Aguiar‐Oliveira et al., 2010). These findings suggest that differences in miRNA signatures can be present between the two syndromes.

## 4 EXPERIMENTAL PROCEDURES

### 4.1 Patient and clinical characteristics

For this study, IGHD (*n* = 23) and control (*n* = 23) subjects matched by age and sex were recruited by ads placed in the local Dwarfs Association building and by word of mouth among the inhabitants of Itabaianinha, SE, Brazil. The Federal University of Sergipe Institutional Review Board approved this study, and all subjects gave informed consent. Inclusion criteria for the IGHD group were short stature and confirmed genotype for GHRHR homozygous c.57+1 A→ G mutation. Individuals of normal height and homozygous genotype to the wild type GHRHR allele were recruited for the control group. Exclusion criteria were age <30 years and previous GH treatment. Blood samples were collected after overnight fasting and centrifuged for serum separation. Serum samples were stored at −80°C for RNA extraction.

A complete physical examination was performed, body weight and height were measured, and body mass index (BMI) was calculated. Blood pressure was the average from three measurements by one physician. In the serum samples, glucose, hemoglobin A1C, total and HDL cholesterol, triglycerides, and creatinine were measured by standard techniques. The LDL concentration was estimated using the Friedwald equation (Friedewald et al., 1972). An oral glucose tolerance test was also performed, with a glucose dose of 1.75 g/kg for IGHD subjects. The Framingham risk score was calculated as described (D'Agostino et al., 2001).

For serum parameters and miRNAs analysis, subjects were divided into two age groups of approximately the same size 32–45 years and 45–82 years, which will hereafter be referred to as young and old, respectively. Statistical analysis of clinical data was performed on SAS Studio (SAS OnDemand for Academics, SAS) using the MIXED procedure to test the effects of genotype, age, or genotype by age interaction. When significant effects were found, the Tukey adjustment was used to correct for multiple comparisons. A value of *p* < 0.05 was considered as significant.

### 4.2 RNA extraction and miRNA library preparation

RNA extraction from 400 μl of serum samples was performed using the miRNEasy Serum/Plasma kit (Qiagen) following manufacturer instructions. MicroRNA libraries from total RNA were prepared using the NEBNEXT Small RNA Library Prep Set for Illumina (New England Biolabs) following the adjusted manufacturer's instructions (Matkovich et al., 2013; Schneider et al., 2018). Briefly, small RNAs from 1 μg of total RNA were ligated with 3′ and 5′ adapters, followed by reverse transcription to produce single‐stranded cDNA. Samples were then amplified by PCR in 14 cycles using different indexes to tag individual samples allowing libraries to be processed in a single flow cell lane during sequencing. The amplified libraries were size‐selected and purified in a 6% acrylamide gel.

The quantity and quality of miRNA libraries were determined using BioAnalyzer and RNA Nano Lab Chip Kit (Agilent Technologies). The samples were combined in a single microtube and submitted to sequencing on a HiSeq 2500 instrument (Illumina Inc.).

### 4.3 miRNAs libraries processing and statistical analyses

All RNA‐Seq data are available at the Sequence Read Archive (SRA) at NCBI under accession number PRJNA724812. Alignment and quantification of miRNA libraries were performed using Cutadapt and bowtie2 (Trapnell et al., 2010). An index of the human and mouse mature miRNAs was built with bowtie2 using miRbase 22, genome build GRCh38 for humans and GRCm38 for mouse (Kozomara & Griffiths‐Jones, 2014). Statistical analyses of differentially expressed miRNAs were performed using linear modeling implemented with the edgeR and limma packages (Robinson et al., 2010) in the R (3.2.2) environment. The edgeR tool implements a range of statistical methodology based on the negative binomial distributions, including empirical Bayes estimation, exact tests, generalized linear models, and quasi‐likelihood tests (McCarthy et al., 2012; Robinson et al., 2010). The count data generated with edgeR were stringently filtered to only keep features with more than 100 counts per million reads in at least 17 samples from one of the groups, then subjected to quantile normalization using the limma:voom function. The voom function from the limma package transforms the count data produced by edgeR into log2‐counts per million (logCPM), then estimates the mean–variance relationship and uses this to compute appropriate observation‐level weights (Law et al., 2014). This process allows using the data for complex model specification where multiple categorical and continuous variables could be included, therefore permitting adjustments for potential confounding effects. Furthermore, the empirical Bayes smoothing of gene‐wise standard deviations provides increased power. Our linear models accounted for the interaction between genotype and age (Genotype‐by‐Age interaction). Gender, BMI, type 2 diabetes, and the Framingham Risk Score were a priori selected as cofactors or covariates to account for potential confounding effects of potential associations between these variables, the miRNA levels and the aging process. The Framingham Score, in particular, was included in the models to efficiently account (with a single additional variable) for the potential confounding effect of multifactorial interactions taking place over time among multiple risk factors to produce cardiovascular disease (D'Agostino et al., 2013). MicroRNAs with an FDR < 0.1 and FC > 3.0 were considered up‐regulated, and FDR < 0.1 and FC < 0.33 were considered down‐regulated. Principal component analysis was also performed using R software.

The same processing and similar statistical analysis procedure were used to analyze raw data from a previously published paper from our group with GH‐deficient female mice (Victoria et al., 2015), except no covariates were included in the model. The samples corresponded to miRNAs extracted from serum of young (6‐month‐old, WT, *n* = 5; df/df, *n* = 5) and old mice (22‐month‐old, WT, *n* = 5; df/df, *n* = 5) with GH deficiency and control littermates.

### 4.4 miRNAs target prediction and enriched pathways and GO Terms

MicroRNAs families were determined using the miRbase Converter package in R (Xu et al., 2018). The mirPath tool (version 3.0) was used to predict target genes of the differentially regulated miRNAs using the microT‐CDS v. 5.0 database (Vlachos et al., 2015) and for retrieving KEGG molecular pathways (Kanehisa & Goto, 2000; Kanehisa et al., 2016), considering *p* values < 0.05 as significant for pathway enrichment. For pathway analysis, the miRNAs were grouped as down‐ or up‐regulated. The MIENTURNET web tool was used to construct miRNA–gene interaction networks (Licursi et al., 2019). TAM 2.0 was used to predict overrepresented miRNA‐function sets (Li et al., 2018).

### 4.5 Cell culture and miRNA mimic transfection

Cell culture was performed to validate the effects of regulated miRNAs on gene expression. Human umbilical vein endothelial cells (HUVECs) were purchased from ATCC (Manassas, Virginia) and cultured in Endothelial medium (R&D Systems) supplemented with endothelial cell growth supplement (R&D Systems), 100 units/ml penicillin, and 100 µg/ml streptomycin (Gibco) in a relative humidity of 95%, 5% CO2, and 37°C.

For transfection, the cells were cultured on 12 well plates at 1 × 10^5^ cells per well in endothelial medium with 1% penicillin/streptomycin and serum free. HUVECs were transfected with 3nM miR‐100‐5p mimic, miR‐195‐5p mimic, miR‐181b‐5p mimic, and miR‐30e‐5p mimics individually or combined (Dharmacon). These miRNAs mimics were selected based on significant miRNAs regulated in serum of IGHD patients. HiPerFect (Qiagen) was used as the transfection reagent, and the manufacturer's protocol was followed as indicated. Transfection was validated after 72 h by assessing the levels of the transfected miRNA mimics by qPCR (Figure S3).

### 4.6 Cell culture RNA Isolation and Quantitative Real‐Time PCR (qPCR)

Total RNA isolation was performed with miRNeasy Mini Kit (Qiagen) in accordance with the manufacturer's instruction. RNA concentration and purity were determined using a plate reader (EpochTM Microplate Spectrophotometer, BioTek). For miRNA quantification, a total of 5 ng of RNA were converted into complementary DNA (cDNA) using TaqMan® Advanced miRNA Assays (Applied BiosystemsTM) according to the manufacturer's protocol. The cDNA samples were diluted ten times as the manufacturer's protocol and stored at −20°C. To verify the relative expression of miR‐100‐5p, miR‐195‐5p, miR‐181b‐5p and miR‐30e‐5p, reactions were performed in duplicate by adding 5 µl of TaqMan® Fast Advanced Master Mix (2×) (Applied BiosystemsTM), 0.5 µl of TaqMan® Advanced miRNA Assay (20×), 2 µl of RNase‐free water, and 2.5 µl of diluted cDNA per well in a MicroAmp® Fast Optical 96‐well reaction plate (Applied Biosystems). The miR‐16‐5p was used as the housekeeping miRNA to normalize qPCR data. Relative miRNA expression was calculated using the 2‐ΔΔCt method.

To investigate mRNA expression, total RNA was converted to cDNA using iScript cDNA synthesis kit (Bio‐Rad) according to the manufacturer's protocol. Reactions were set up in a MicroAmp® Fast Optical 96‐well reaction plate with 2 μl of diluted cDNA, 0.2 μl each of forward and reverse primers given below, 12.6 μl of nuclease‐free water, and 5 μl of Fast SYBR Green Master Mix (Applied Biosystems) per well. To normalize the qPCR data, beta‐2‐microglobulin (B2 M) was selected as the housekeeping gene. The method 2‐ΔΔCt was used for calculating relative expression. The Student's *t* test was performed for statistical analysis.

Primer sequences were as follows (5’ > 3’) AKT‐FW: TCTCGGCTCTTGAGTACTTG, AKT‐REV: TCACTGATGCCCTCTTTGCA, mTOR‐FW: ATGCTTGGAACCGGACCTG, mTOR‐REV: TCTTGACTCATCTCTCGGAGTT, IRS‐1‐FW: TCGAGTACTACGAGAACGAG, IRS1‐REV: TAGAGAGCCACCAGGTGCTT, NFκB‐FW: CTCCGAGACTTTCGAGGAAATAC, NFκB‐REV: GCCATTGTAGTTGGTAGCCTTCA, PPM1B‐FOR: GGGACGTTTGCCCATCTTAT, PPM1B‐REV CAACATGCCTGCCAACTT TATT, IR‐A‐FOR: GTTTTCGTCCCAGCCATC, IR‐A‐REV CCAACATCGCCAAGGGACCT; B2M‐FW: GAGTATGCCTGCCGTGTGAA, B2M‐REV: CGGCATCTTCAAACCTCCAT.

### 4.7 Statistical analysis

Data normality was tested using the Shapiro–Wilk test, and non‐normal data were log‐transformed to approximate normality. Differences in baseline clinical characteristics were assessed using the Welch two‐sample t test (for continuous variables) or the Fisher exact test (for categorical variables). Differential expression analysis of circulating miRNA was performed as described in Section 4.3. Partial correlations between baseline levels of clinical variables and miRNA expression levels adjusting for the potential confounding effects of Gender, BMI, type 2 diabetes, and the Framingham Risk Score were calculated in the R environment. As mentioned in Section 4.3, these variables were a priori selected as cofactors or covariates to account for their potential confounding effects given by potential associations between these variables and both the miRNA levels and the aging process. The Benjamini–Hochberg correction was implemented to account for multiple testing. A correlation network including only significant correlations (*r* > 0.2, FDR < 0.05) between differentially expressed miRNAs and clinical measures was generated using Cytoscape 3.5.1.

## 5 CONCLUSION

We observed significant regulation of age‐related miRNAs in human subjects with untreated lifetime IGHD. These miRNAs have an important overlap with serum‐regulated miRNAs in GH‐deficient df/df mice, which have a remarkable extension of healthspan and lifespan. Importantly, predicted target genes for serum‐regulated miRNAs in IGHD patients contribute to insulin, inflammation, and aging‐related pathways, such as mTOR and FoxO pathways. These main up‐regulated aging‐related miRNAs, miR‐100‐5p, miR‐195‐5p, miR‐181b‐5p, and miR‐30e‐5p, were found to regulate in vitro expression of the age‐related genes mTOR, AKT, NFκB, and IRS1.

## CONFLICT OF INTEREST

The authors declare no conflict of interest.

## AUTHOR CONTRIBUTIONS

TDS performed experiments and wrote the manuscript; AS performed RNA‐Seq analysis and wrote the manuscript; CGM, CRPO, and AAOS performed sample and data collection from patients; ADCN and SN performed cell culture experiments; JD, YONL, and GL performed RNA‐Seq and statistical analysis; RS, NM, and AB involved in conceptualization of the idea and data interpretation; MHAO and MMM involved in conceptualization and coordination of experiments and data interpretation. All authors critically revised the manuscript for important intellectual content and approved the final manuscript.

## Supporting information

Supplementary Material

## Data Availability

The data that support the findings of this study will be available from the corresponding author upon reasonable request.
