# Supplementary material for: Circulating microRNA profile in humans and mice with congenital GH deficiency
Source: Aging Cell. 2021 Jun 12;20(7):e13420. doi: 10.1111/acel.13420 (PMC8282278; doi:10.1111/acel.13420)
Supplement: Supplementary file 1 — Supplementary Material [file ACEL-20-e13420-s001.docx]

**Circulating microRNA profile in humans and mice with congenital GH deficiency**

**Tatiana D. Saccon^1*^, Augusto Schneider^2*^, Cindi G. Marinho^3^, Allancer D. C. Nunes^4^, Sarah Noureddine^4^, Joseph Dhahbi^5^, Yury O. Nunez Lopez^6^, Gage LeMunyan^5^, Roberto Salvatori^7^, Carla R. P. Oliveira^3^, Alécia A. Oliveira-Santos^3^, Nicolas Musi^8^, Andrzej Bartke^9^, Manuel H. Aguiar-Oliveira^3#^, Michal M. Masternak^4#^**

1 Centro de Desenvolvimento Tecnológico, Universidade Federal de Pelotas, Pelotas, Brazil,

2 Faculdade de Nutrição, Universidade Federal de Pelotas, Pelotas, Brazil,

3 Division of Endocrinology, Health Sciences Graduate Program, Federal University of Sergipe 49060–100, Aracaju, Sergipe, Brazil.

4 Burnett School of Biomedical Sciences, College of Medicine, University of Central Florida, Orlando, FL, United States

5Departmentof Medical Education, School of Medicine, California University of Science & Medicine, San Bernardino, CA, United States

6Translational Research Institute for Metabolism and Diabetes, Advent Health, Orlando, FL, United States,

7Division of Endocrinology, Diabetes and Metabolism, Department of Medicine, The Johns Hopkins University School of Medicine Baltimore, Maryland 21287 USA

8Barshop Institute for Longevity and Aging Studies, Center for Healthy Aging, University of Texas Health Sciences Center at San Antonio and South Texas Veterans Health Care System, San Antonio, TX 78229, United States; San Antonio Geriatric Research, Education and Clinical Center, South Texas Veterans Health Care System, San Antonio, TX 78229, United States

9Southern Illinois University School of Medicine, Department of Internal Medicine, 801 N. Rutledge, P.O. Box 19628, Springfield, Illinois 62702, USA

*Both authors contributed equally to this paper

#Both authors contributed equally to this paper

**#Corresponding authors:**

**Michal M. Masternak**

University of Central Florida, Burnett School of Biomedical Sciences College of Medicine

6900 Lake Nona Blvd, Orlando, FL 32827, USA.

E-mail: michal.masternak@ucf.edu

**Manuel H. Aguiar-Oliveira**

Division of Endocrinology, Health Sciences Graduate Program, Federal University of Sergipe 49060–100, Aracaju, Sergipe, Brazil

E-mail: herminio.endo@gmail.com

**Supplementary Figures**

**
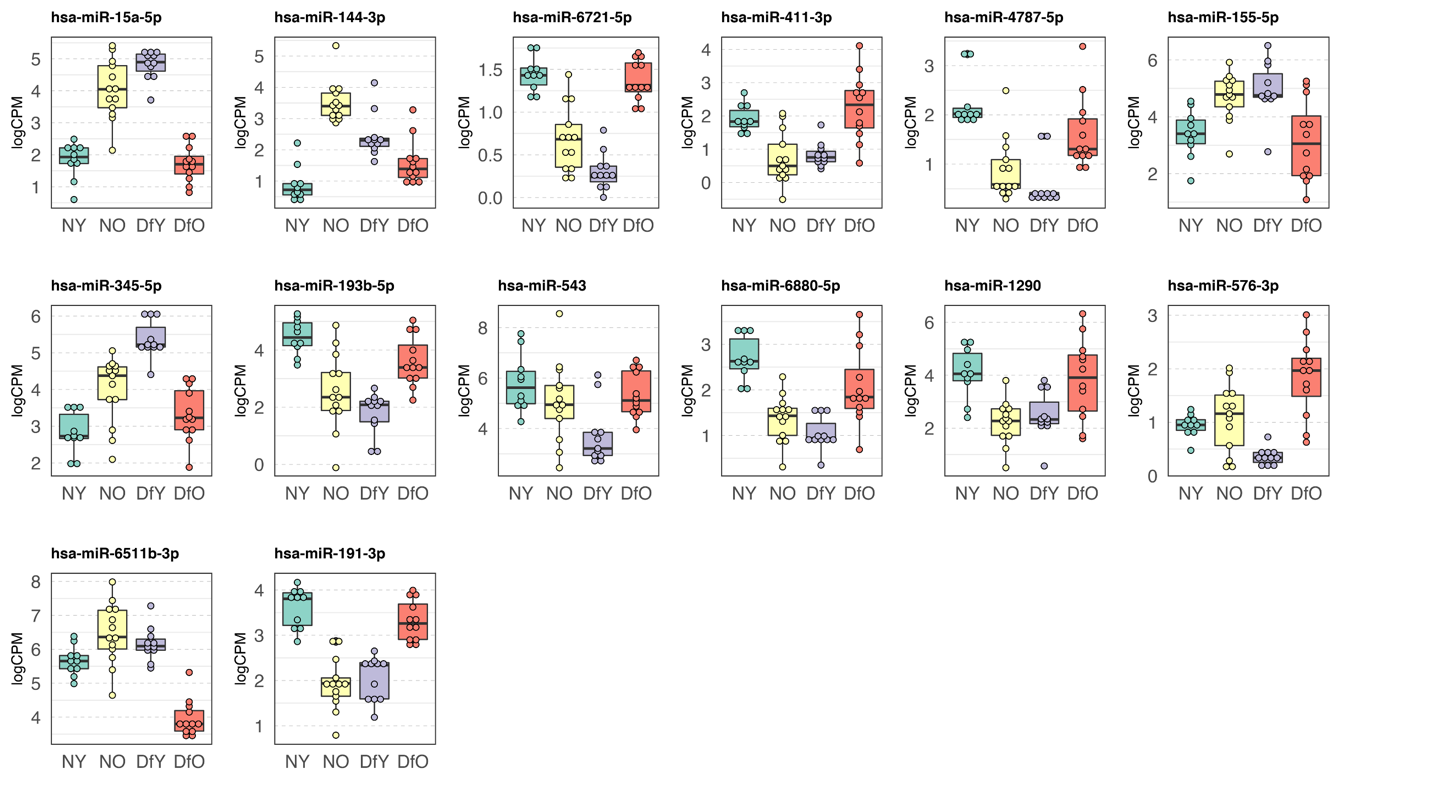
Suppl. Figure 1 -** Boxplot of regulated microRNAs (miRNAs) with a genotype by age interaction in the serum of IGHD and control subjects.

**
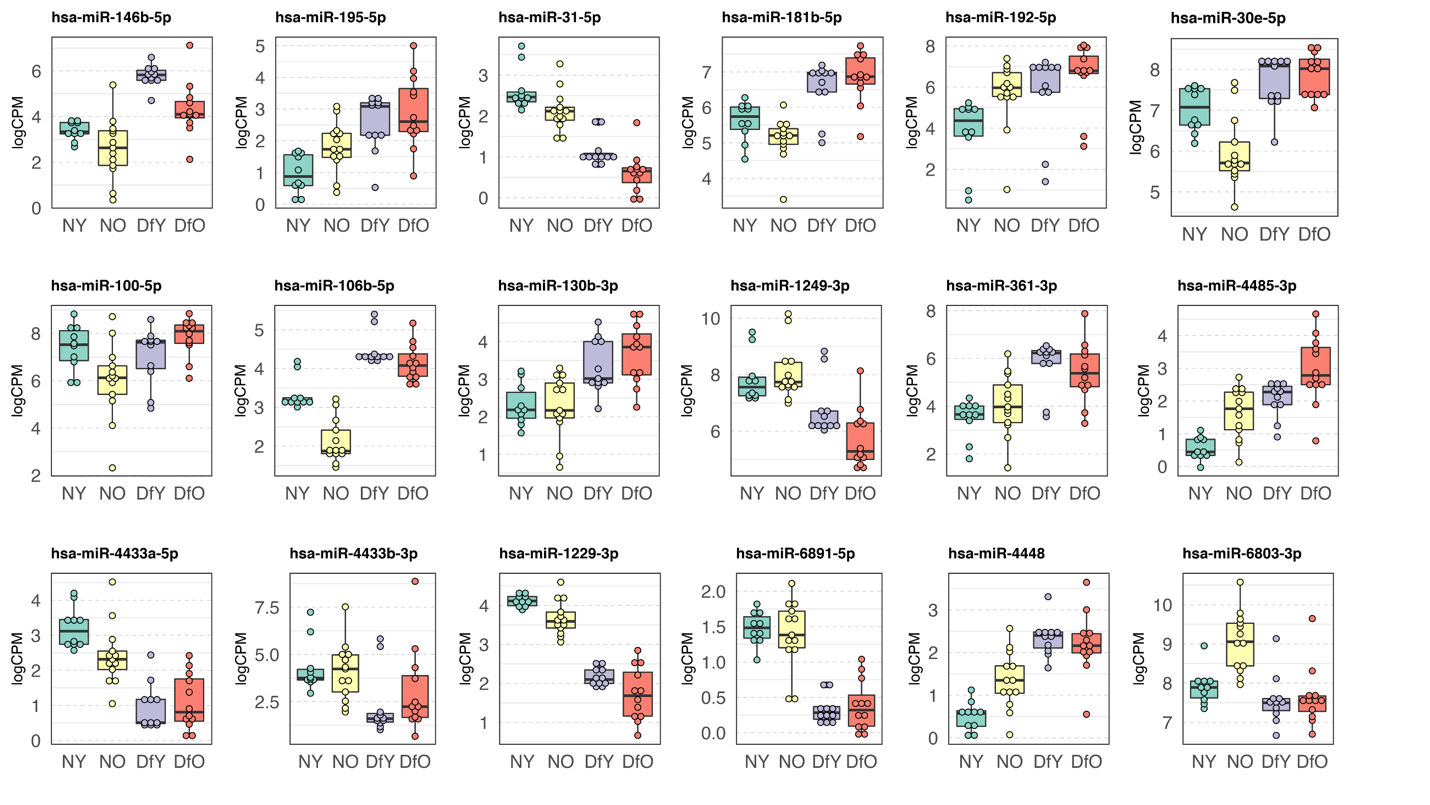
**

**Suppl. Figure 2 -** Boxplot of regulated microRNAs (miRNAs) with a genotype effect in the serum of IGHD and control subjects.


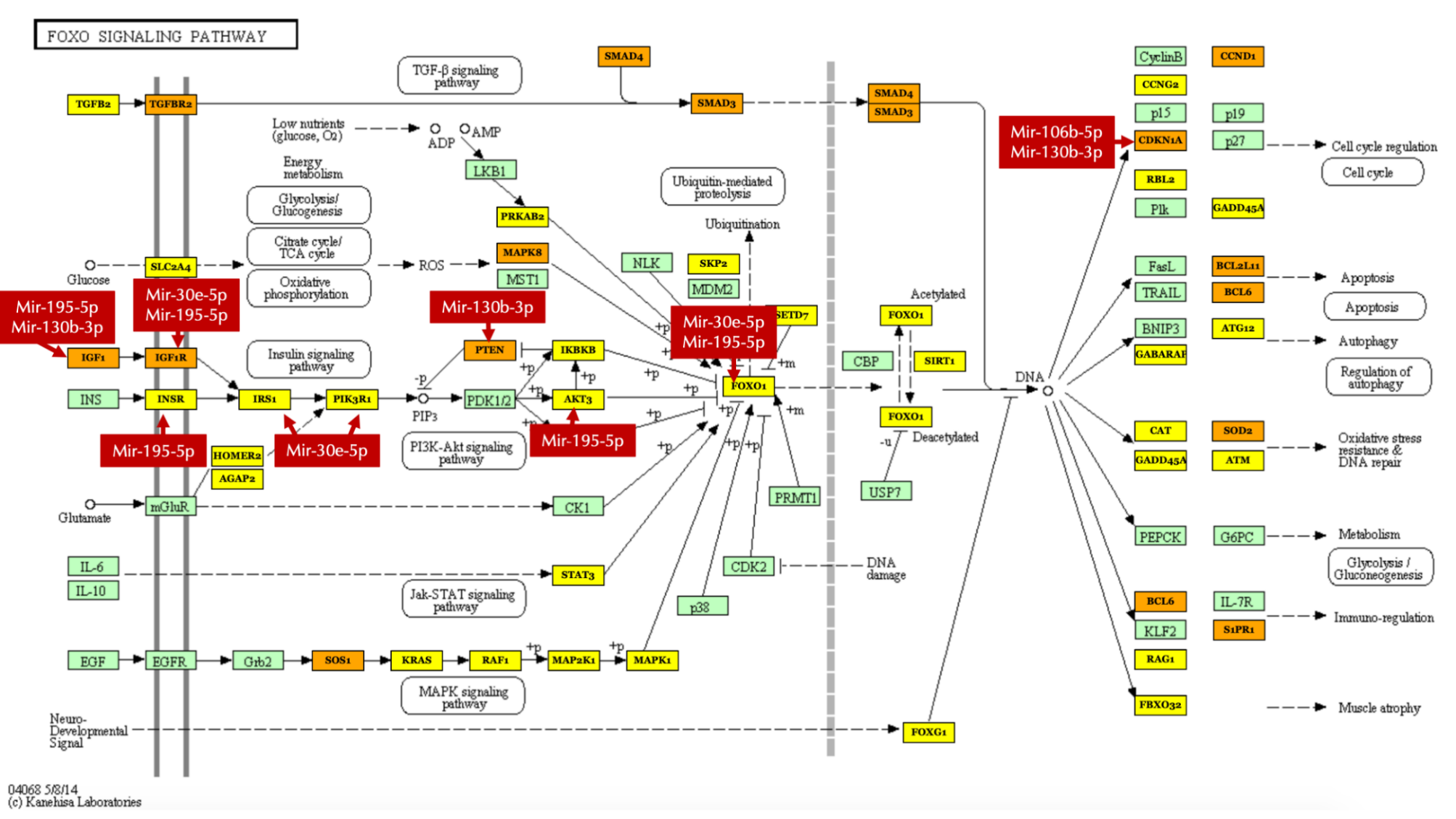
**Suppl. Figure 3 -** Schematic representation of predicted regulated target genes and associated miRNAs in the KEGG FoxO signaling pathway for IGHD subjects. Green boxes indicate non-regulated genes; yellow boxes indicate a gene predicted to be regulated by only one miRNA; orange boxes indicate a gene predicted to be regulated by more than one miRNA. Some miRNAs are highlighted in red boxes and associated to its respective predicted to be target genes. Images generated using the mirPath tool (version 3.0) and the microT-CDS v. 5.0 database


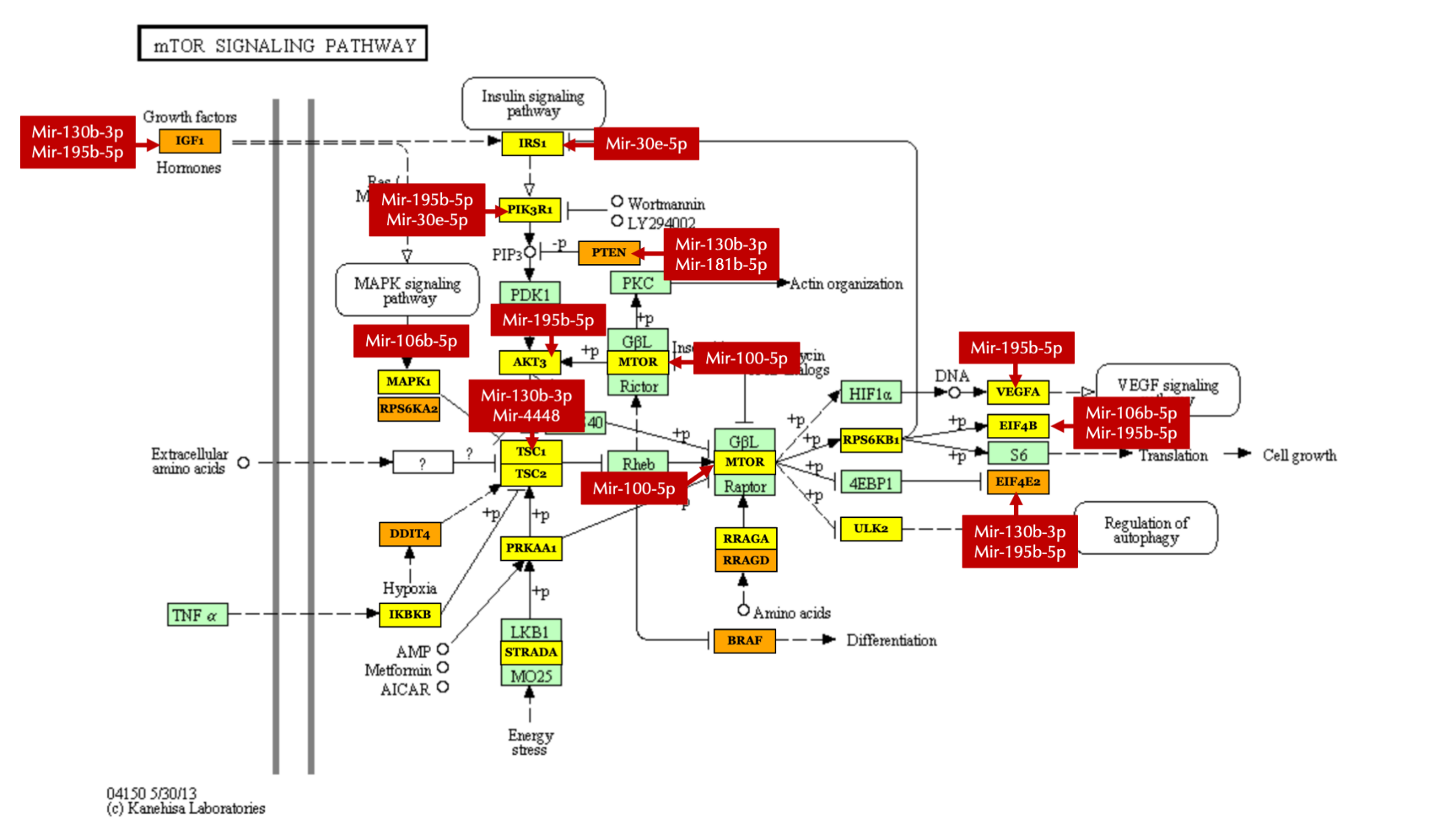


**Suppl. Figure 4 -** Schematic representation of predicted regulated target genes and associated miRNAs in the KEGG mTOR signaling pathway for IGHD subjects. Green boxes indicate non-regulated genes; yellow boxes indicate a gene predicted to be regulated by only one miRNA; orange boxes indicate a gene predicted to be regulated by more than one miRNA. Some miRNAs are highlighted in red boxes and associated to its respective predicted to be target genes. Images generated using the mirPath tool (version 3.0) and the microT-CDS v. 5.0 database


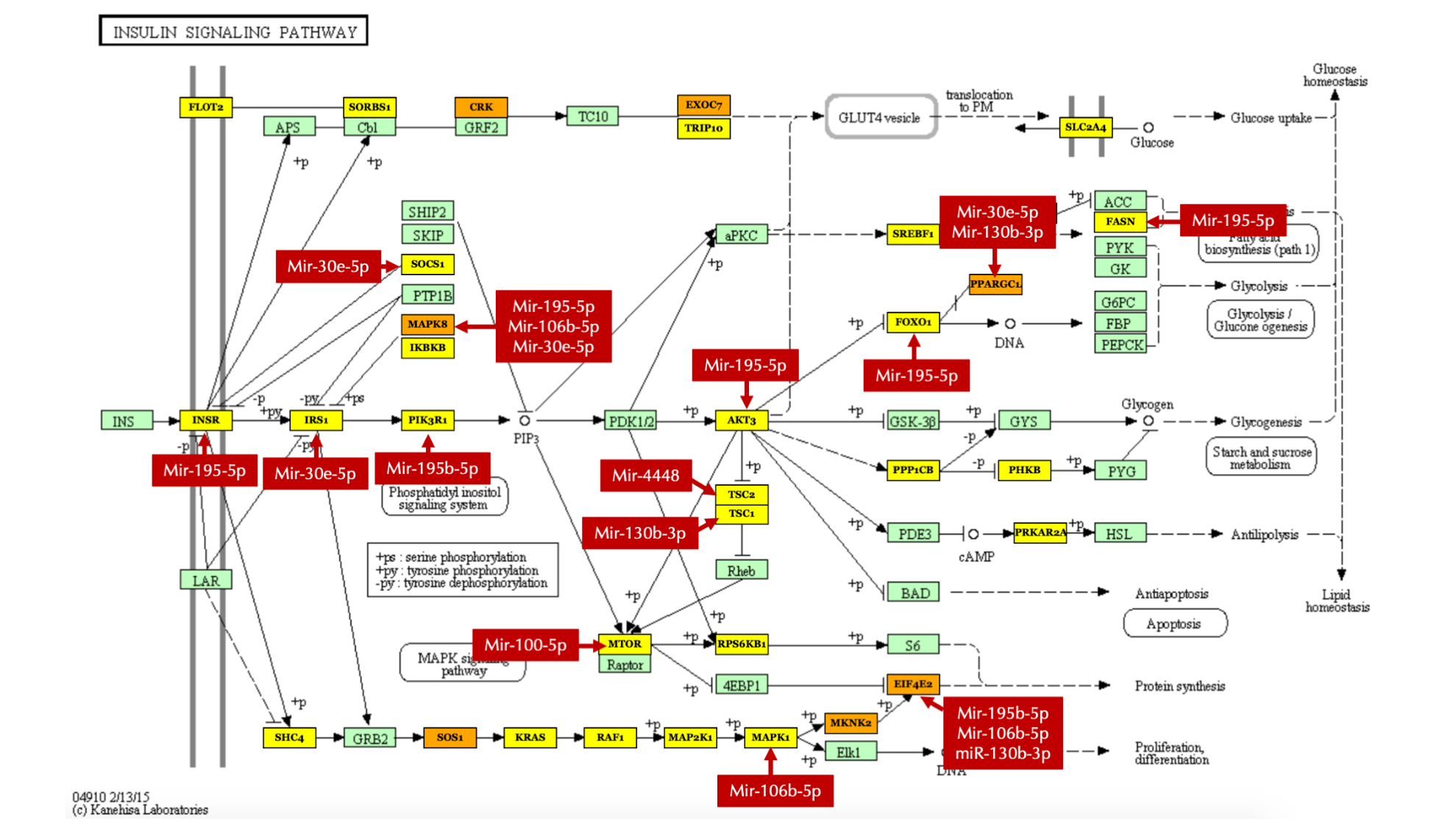


**Suppl. Figure 5 -** Schematic representation of predicted regulated target genes and associated miRNAs in the KEGG insulin signaling pathway for IGHD subjects. Green boxes indicate non-regulated genes; yellow boxes indicate a gene predicted to be regulated by only one miRNA; orange boxes indicate a gene predicted to be regulated by more than one miRNA. Some miRNAs are highlighted in red boxes and associated to its respective predicted to be target genes. Images generated using the mirPath tool (version 3.0) and the microT-CDS v. 5.0 database

**
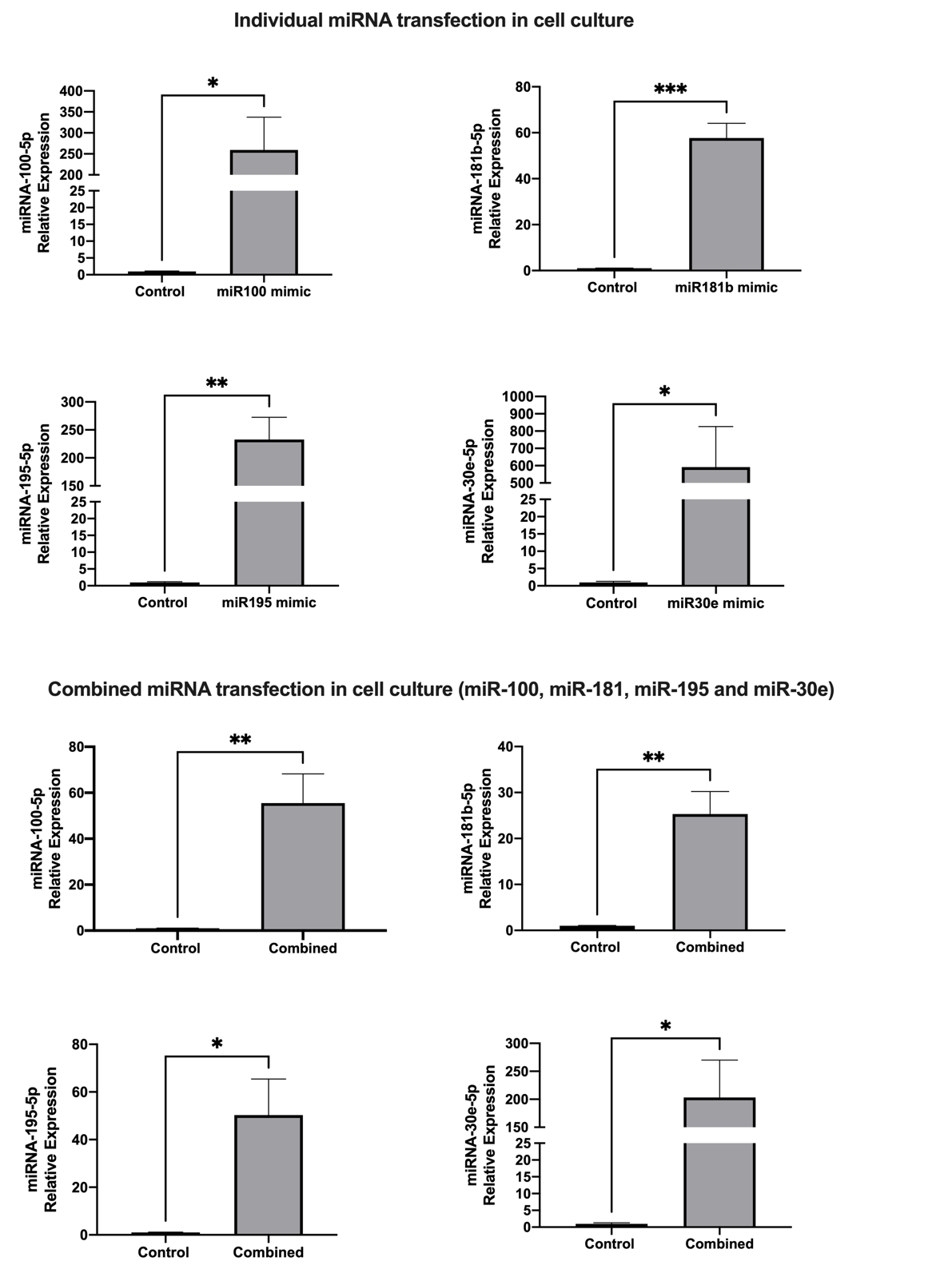
**

**Suppl. Figure 6 -** Relative expression of miR-100-5p, miR-195-5p, miR-181b-5p, and miR-30e-5p individually from in vitro HUVEC cells transfected with 3nM of the same miRNAs individually or in combination as a mix.


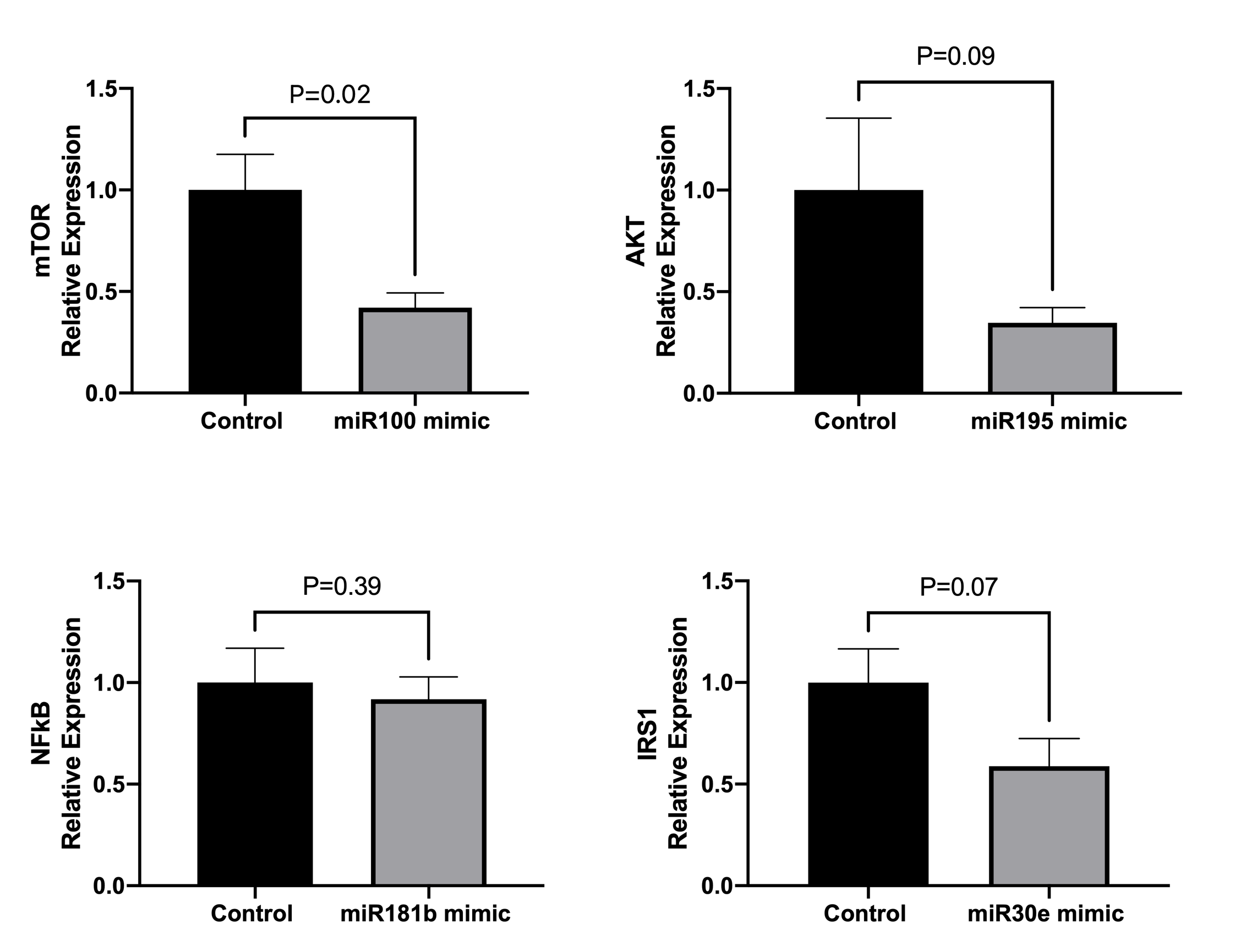


**Suppl. Figure 7 -** Relative expression of predicted target genes (mTOR, AKT, NFκB, and IRS1) from in vitro HUVEC cells transfected with 3nM of miR-100-5p, miR-195-5p, miR-181b-5p, and miR-30e-5p individually.


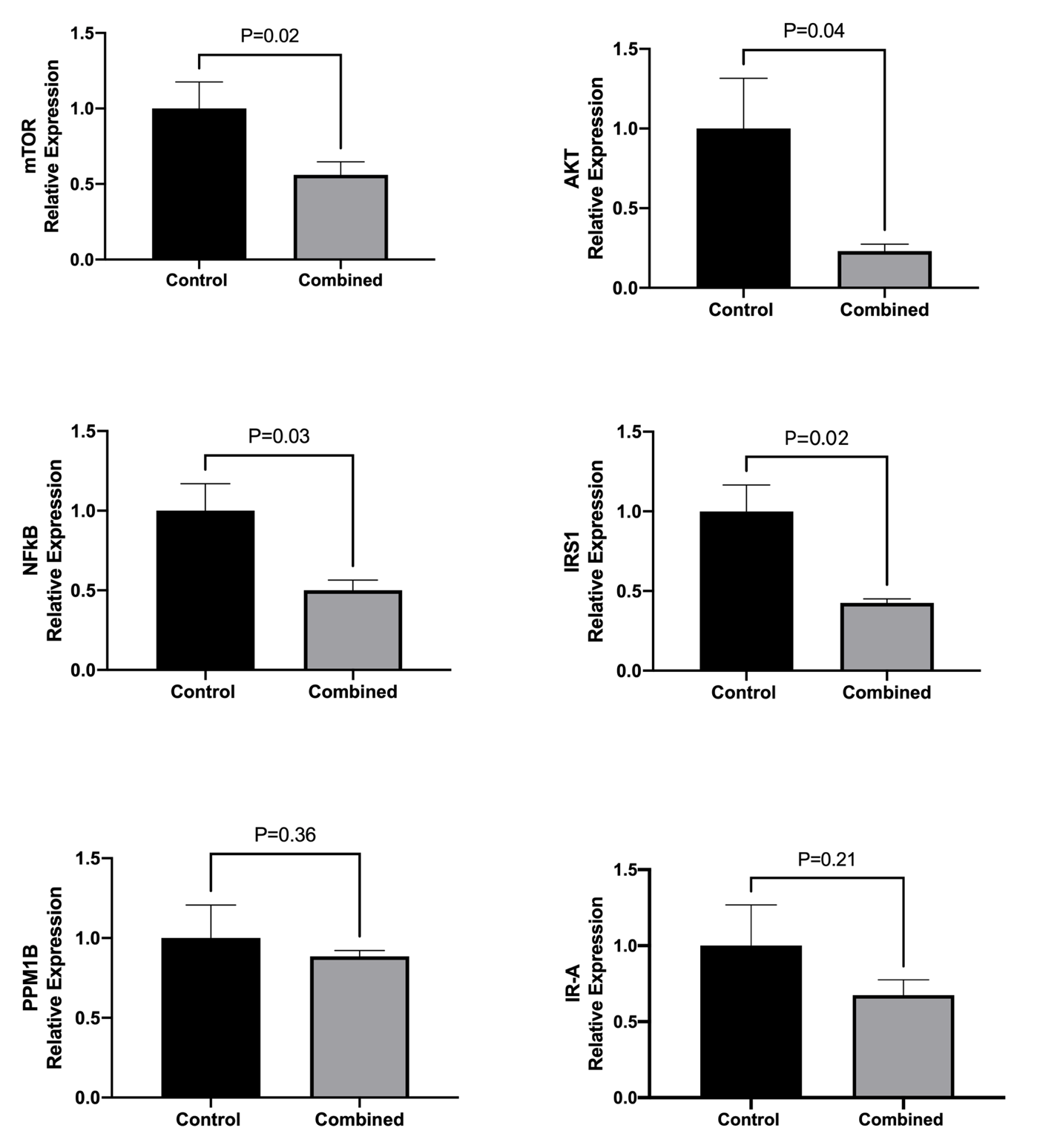


**Suppl. Figure 8 -** Relative expression of predicted target genes (mTOR, AKT, NFκB, and IRS1) and controls (PP1MB and IR-A) from in vitro HUVEC cells transfected with 3nM of miR-100-5p, miR-195-5p, miR-181b-5p, and miR-30e-5p combined in a single mix.

**Supplementary Tables**

**Suppl. Table 1** – Correlation between miRNAs and clinical parameters in IGHD individuals

| **miRNA** | **Clinical Parameter** | **Correlation** | **P value** | **FDR** |
| --- | --- | --- | --- | --- |
| hsa-miR-100-5p | BP mean (mm Hg) Diastolic | 0.31 | 0.034 | 0.050 |
| hsa-miR-106b-5p | BP mean (mm Hg) Diastolic | -0.36 | 0.013 | 0.023 |
| hsa-miR-1229-3p | BP mean (mm Hg) Diastolic | 0.31 | 0.039 | 0.055 |
| hsa-miR-130b-3p | BP mean (mm Hg) Diastolic | -0.31 | 0.036 | 0.052 |
| hsa-miR-142-3p | BP mean (mm Hg) Diastolic | -0.51 | 0.000 | 0.001 |
| hsa-miR-146b-5p | BP mean (mm Hg) Diastolic | -0.30 | 0.046 | 0.063 |
| hsa-miR-148b-3p | BP mean (mm Hg) Diastolic | -0.39 | 0.007 | 0.014 |
| hsa-miR-191-3p | BP mean (mm Hg) Diastolic | 0.43 | 0.003 | 0.007 |
| hsa-miR-193b-5p | BP mean (mm Hg) Diastolic | 0.41 | 0.004 | 0.009 |
| hsa-miR-31-5p | BP mean (mm Hg) Diastolic | 0.42 | 0.003 | 0.007 |
| hsa-miR-345-5p | BP mean (mm Hg) Diastolic | -0.43 | 0.003 | 0.006 |
| hsa-miR-370-3p | BP mean (mm Hg) Diastolic | 0.34 | 0.022 | 0.035 |
| hsa-miR-374a-5p | BP mean (mm Hg) Diastolic | -0.31 | 0.039 | 0.056 |
| hsa-miR-4448 | BP mean (mm Hg) Diastolic | -0.35 | 0.018 | 0.030 |
| hsa-miR-4732-5p | BP mean (mm Hg) Diastolic | -0.38 | 0.009 | 0.017 |
| hsa-miR-500a-3p | BP mean (mm Hg) Diastolic | 0.40 | 0.007 | 0.013 |
| hsa-miR-6796-5p | BP mean (mm Hg) Diastolic | 0.36 | 0.014 | 0.025 |
| hsa-miR-6891-5p | BP mean (mm Hg) Diastolic | 0.29 | 0.049 | 0.066 |
| hsa-miR-877-3p | BP mean (mm Hg) Diastolic | -0.53 | 0.000 | 0.001 |
| hsa-miR-106b-5p | BP mean (mm Hg) Systolic | -0.45 | 0.002 | 0.004 |
| hsa-miR-142-3p | BP mean (mm Hg) Systolic | -0.41 | 0.004 | 0.009 |
| hsa-miR-146b-5p | BP mean (mm Hg) Systolic | -0.63 | 0.000 | 0.000 |
| hsa-miR-193b-5p | BP mean (mm Hg) Systolic | 0.38 | 0.008 | 0.016 |
| hsa-miR-345-5p | BP mean (mm Hg) Systolic | -0.40 | 0.006 | 0.012 |
| hsa-miR-374a-5p | BP mean (mm Hg) Systolic | -0.53 | 0.000 | 0.001 |
| hsa-miR-411-3p | BP mean (mm Hg) Systolic | 0.41 | 0.004 | 0.009 |
| hsa-miR-500a-3p | BP mean (mm Hg) Systolic | 0.41 | 0.005 | 0.010 |
| hsa-miR-589-3p | BP mean (mm Hg) Systolic | 0.45 | 0.002 | 0.005 |
| hsa-miR-664b-5p | BP mean (mm Hg) Systolic | 0.36 | 0.015 | 0.026 |
| hsa-miR-7114-3p | BP mean (mm Hg) Systolic | 0.48 | 0.001 | 0.002 |
| hsa-miR-199a-5p | LDL | 0.37 | 0.012 | 0.020 |
| hsa-miR-215-5p | LDL | 0.31 | 0.036 | 0.052 |
| hsa-miR-378a-3p | LDL | 0.47 | 0.001 | 0.003 |
| hsa-miR-433-3p | LDL | -0.33 | 0.027 | 0.041 |
| hsa-miR-4433a-3p | LDL | -0.30 | 0.045 | 0.062 |
| hsa-miR-500a-3p | LDL | 0.36 | 0.014 | 0.025 |
| hsa-miR-8485 | LDL | -0.33 | 0.023 | 0.036 |
| hsa-miR-941 | LDL | 0.34 | 0.022 | 0.035 |
| hsa-miR-1229-3p | Creatinine | 0.38 | 0.009 | 0.016 |
| hsa-miR-1293 | Creatinine | -0.32 | 0.033 | 0.048 |
| hsa-miR-130b-3p | Creatinine | -0.57 | 0.000 | 0.000 |
| hsa-miR-146b-5p | Creatinine | -0.29 | 0.049 | 0.066 |
| hsa-miR-193b-5p | Creatinine | 0.30 | 0.043 | 0.060 |
| hsa-miR-199a-5p | Creatinine | 0.31 | 0.039 | 0.056 |
| hsa-miR-29b-1-5p | Creatinine | -0.44 | 0.002 | 0.006 |
| hsa-miR-31-5p | Creatinine | 0.38 | 0.009 | 0.016 |
| hsa-miR-433-3p | Creatinine | -0.44 | 0.002 | 0.005 |
| hsa-miR-576-3p | Creatinine | -0.31 | 0.034 | 0.050 |
| hsa-miR-654-3p | Creatinine | 0.34 | 0.021 | 0.034 |
| hsa-miR-6734-5p | Creatinine | -0.56 | 0.000 | 0.000 |
| hsa-miR-130b-3p | FBG | -0.31 | 0.038 | 0.055 |
| hsa-miR-193b-5p | FBG | 0.39 | 0.007 | 0.014 |
| hsa-miR-199a-5p | FBG | -0.35 | 0.017 | 0.029 |
| hsa-miR-345-5p | FBG | -0.36 | 0.015 | 0.025 |
| hsa-miR-378a-3p | FBG | -0.35 | 0.017 | 0.028 |
| hsa-miR-4732-5p | FBG | -0.40 | 0.006 | 0.012 |
| hsa-miR-654-3p | FBG | 0.43 | 0.003 | 0.007 |
| hsa-miR-8485 | FBG | 0.36 | 0.013 | 0.023 |
| hsa-miR-1290 | GTT | 0.33 | 0.027 | 0.041 |
| hsa-miR-193b-5p | GTT | 0.41 | 0.006 | 0.011 |
| hsa-miR-199a-5p | GTT | -0.47 | 0.001 | 0.003 |
| hsa-miR-29b-1-5p | GTT | 0.30 | 0.043 | 0.060 |
| hsa-miR-345-5p | GTT | -0.34 | 0.024 | 0.037 |
| hsa-miR-374a-5p | GTT | -0.31 | 0.037 | 0.053 |
| hsa-miR-411-3p | GTT | 0.36 | 0.015 | 0.025 |
| hsa-miR-4732-5p | GTT | -0.35 | 0.020 | 0.032 |
| hsa-miR-5787 | GTT | -0.30 | 0.049 | 0.066 |
| hsa-miR-654-3p | GTT | 0.35 | 0.020 | 0.033 |
| hsa-miR-192-5p | HbA1c | 0.33 | 0.025 | 0.038 |
| hsa-miR-193b-5p | HbA1c | 0.36 | 0.015 | 0.026 |
| hsa-miR-199a-5p | HbA1c | -0.47 | 0.001 | 0.002 |
| hsa-miR-30e-5p | HbA1c | 0.29 | 0.048 | 0.066 |
| hsa-miR-31-5p | HbA1c | -0.33 | 0.023 | 0.036 |
| hsa-miR-335-3p | HbA1c | 0.34 | 0.019 | 0.031 |
| hsa-miR-361-3p | HbA1c | 0.41 | 0.005 | 0.010 |
| hsa-miR-4732-5p | HbA1c | -0.32 | 0.031 | 0.047 |
| hsa-miR-654-3p | HbA1c | 0.34 | 0.022 | 0.035 |
| hsa-miR-6796-5p | HbA1c | -0.31 | 0.034 | 0.050 |
| hsa-miR-8485 | HbA1c | 0.30 | 0.041 | 0.058 |
| hsa-miR-5787 | HDL | -0.31 | 0.038 | 0.055 |
| hsa-miR-100-5p | Triglycerides | 0.50 | 0.000 | 0.001 |
| hsa-miR-139-3p | Triglycerides | 0.45 | 0.002 | 0.004 |
| hsa-miR-139-5p | Triglycerides | 0.42 | 0.003 | 0.007 |
| hsa-miR-191-3p | Triglycerides | 0.45 | 0.002 | 0.004 |
| hsa-miR-193b-5p | Triglycerides | 0.47 | 0.001 | 0.003 |
| hsa-miR-214-3p | Triglycerides | 0.42 | 0.003 | 0.007 |
| hsa-miR-3074-5p | Triglycerides | -0.35 | 0.018 | 0.030 |
| hsa-miR-30e-5p | Triglycerides | 0.35 | 0.017 | 0.029 |
| hsa-miR-345-5p | Triglycerides | -0.29 | 0.048 | 0.065 |
| hsa-miR-374a-5p | Triglycerides | -0.41 | 0.005 | 0.010 |
| hsa-miR-411-3p | Triglycerides | 0.42 | 0.004 | 0.009 |
| hsa-miR-433-3p | Triglycerides | -0.34 | 0.020 | 0.033 |
| hsa-miR-642a-3p | Triglycerides | 0.34 | 0.022 | 0.035 |
| hsa-miR-6511b-3p | Triglycerides | -0.34 | 0.022 | 0.035 |
| hsa-miR-652-3p | Triglycerides | 0.41 | 0.005 | 0.011 |
| hsa-miR-7114-3p | Triglycerides | 0.55 | 0.000 | 0.000 |
| hsa-miR-877-5p | Triglycerides | 0.34 | 0.019 | 0.031 |
| hsa-miR-96-5p | Triglycerides | 0.33 | 0.023 | 0.036 |
| hsa-miR-100-5p | Total cholesterol | 0.33 | 0.026 | 0.040 |
| hsa-miR-139-5p | Total cholesterol | 0.30 | 0.045 | 0.063 |
| hsa-miR-199a-5p | Total cholesterol | 0.36 | 0.015 | 0.026 |
| hsa-miR-214-3p | Total cholesterol | 0.34 | 0.023 | 0.036 |
| hsa-miR-374a-5p | Total cholesterol | -0.34 | 0.021 | 0.034 |
| hsa-miR-378a-3p | Total cholesterol | 0.44 | 0.002 | 0.005 |
| hsa-miR-433-3p | Total cholesterol | -0.35 | 0.017 | 0.029 |
| hsa-miR-4433a-3p | Total cholesterol | -0.29 | 0.050 | 0.068 |
| hsa-miR-500a-3p | Total cholesterol | 0.34 | 0.022 | 0.035 |
| hsa-miR-7114-3p | Total cholesterol | 0.30 | 0.045 | 0.062 |
| hsa-miR-8485 | Total cholesterol | -0.32 | 0.028 | 0.043 |
| hsa-miR-941 | Total cholesterol | 0.31 | 0.034 | 0.050 |

**Suppl. Table 1** – miRNAs regulated with a genotype by age interaction in GH-deficient Ames dwarf mice

| **miRNAs** | **logFC** | **logCPM** | **LR** | **PValue** | **FDR** |
| --- | --- | --- | --- | --- | --- |
| mmu-miR-34c-3p | 5.77 | 2.97 | 23.52 | 1.24E-06 | 3.67E-04 |
| mmu-miR-34c-5p | 4.79 | 5.59 | 20.82 | 5.04E-06 | 0.001 |
| mmu-miR-342-3p | -1.79 | 6.72 | 14.15 | 1.69E-04 | 0.014 |
| mmu-miR-541-5p | 4.58 | 7.80 | 13.96 | 1.86E-04 | 0.014 |
| mmu-miR-136-5p | 5.02 | 6.27 | 12.41 | 4.26E-04 | 0.022 |
| mmu-miR-34b-3p | 3.47 | 3.60 | 12.31 | 4.50E-04 | 0.022 |
| mmu-miR-434-5p | 3.80 | 5.93 | 12.05 | 0.001 | 0.022 |
| mmu-miR-127-3p | 3.89 | 10.16 | 11.36 | 0.001 | 0.028 |
| mmu-miR-431-5p | 3.64 | 4.54 | 9.89 | 0.002 | 0.055 |
| mmu-miR-344d-3p | 3.66 | 4.45 | 9.66 | 0.002 | 0.056 |
| mmu-miR-195a-5p | 2.12 | 6.43 | 9.37 | 0.002 | 0.060 |
| mmu-miR-381-3p | 3.99 | 7.32 | 9.17 | 0.002 | 0.061 |
| mmu-miR-1441-3p | 1.12 | 9.99 | 8.94 | 0.003 | 0.064 |
| mmu-miR-155-5p | -2.06 | 4.03 | 8.34 | 0.004 | 0.076 |
| mmu-miR-215-5p | -1.86 | 13.24 | 8.29 | 0.004 | 0.076 |
| mmu-miR-410-3p | 2.66 | 7.02 | 8.23 | 0.004 | 0.076 |
| mmu-miR-150-5p | -1.49 | 11.21 | 7.93 | 0.005 | 0.083 |
| mmu-miR-146a-5p | -2.28 | 8.36 | 7.86 | 0.005 | 0.083 |

**Suppl. Table 2** – miRNAs regulated by genotype in GH-deficient Ames dwarf mice

| **miRNAs** | **logFC** | **logCPM** | **LR** | **PValue** | **FDR** |
| --- | --- | --- | --- | --- | --- |
| **Down-regulated** |  |  |  |  |  |
| mmu-miR-122-5p | -2.41 | 6.62 | 8.59 | 0.003 | 0.032 |
| mmu-miR-381-3p | -2.34 | 7.32 | 8.31 | 0.004 | 0.032 |
| mmu-miR-802-5p | -2.27 | 5.98 | 18.72 | 1.52E-05 | 0.002 |
| mmu-miR-34c-5p | -2.21 | 5.59 | 10.54 | 0.001 | 0.023 |
| mmu-miR-208b-3p | -2.08 | 4.53 | 8.43 | 0.004 | 0.032 |
| mmu-miR-136-5p | -2.06 | 6.27 | 5.11 | 0.024 | 0.091 |
| mmu-miR-411-5p | -1.95 | 5.90 | 4.93 | 0.026 | 0.095 |
| mmu-miR-134-5p | -1.93 | 3.82 | 6.65 | 0.010 | 0.053 |
| mmu-miR-455-5p | -1.81 | 3.79 | 10.21 | 0.001 | 0.023 |
| mmu-miR-410-3p | -1.80 | 7.02 | 9.19 | 0.002 | 0.032 |
| mmu-miR-434-3p | -1.77 | 8.75 | 11.87 | 0.001 | 0.014 |
| mmu-miR-434-5p | -1.76 | 5.93 | 6.16 | 0.013 | 0.064 |
| mmu-miR-127-3p | -1.73 | 10.16 | 5.32 | 0.021 | 0.083 |
| mmu-miR-341-3p | -1.67 | 4.42 | 7.07 | 0.008 | 0.046 |
| mmu-miR-300-3p | -1.59 | 6.37 | 6.31 | 0.012 | 0.060 |
| mmu-miR-6240 | -1.55 | 4.00 | 15.26 | 9.38E-05 | 0.005 |
| mmu-miR-34b-3p | -1.49 | 3.60 | 6.24 | 0.012 | 0.062 |
| mmu-miR-224-5p | -1.34 | 4.47 | 4.98 | 0.026 | 0.094 |
| mmu-miR-194-5p | -1.32 | 5.31 | 15.46 | 8.44E-05 | 0.005 |
| mmu-miR-199a-5p | -1.23 | 5.26 | 4.93 | 0.026 | 0.095 |
| mmu-miR-204-5p | -1.13 | 3.16 | 8.80 | 0.003 | 0.032 |
| mmu-miR-192-5p | -0.97 | 13.96 | 8.87 | 0.003 | 0.032 |
| mmu-miR-101c | -0.91 | 10.95 | 8.03 | 0.005 | 0.033 |
| mmu-miR-101a-3p | -0.91 | 10.95 | 8.03 | 0.005 | 0.033 |
| mmu-miR-193b-3p | -0.90 | 6.03 | 7.14 | 0.008 | 0.046 |
| mmu-miR-143-3p | -0.90 | 12.37 | 5.52 | 0.019 | 0.081 |
| mmu-miR-145a-3p | -0.86 | 4.91 | 5.52 | 0.019 | 0.081 |
| mmu-miR-30a-3p | -0.82 | 8.68 | 7.59 | 0.006 | 0.040 |
| mmu-miR-15a-3p | -0.81 | 5.61 | 12.51 | 4.04E-04 | 0.013 |
| mmu-miR-335-5p | -0.81 | 6.00 | 5.32 | 0.021 | 0.083 |
| mmu-miR-196b-5p | -0.73 | 3.75 | 6.95 | 0.008 | 0.047 |
| mmu-miR-146b-5p | -0.65 | 6.54 | 5.57 | 0.018 | 0.081 |
| mmu-miR-148a-3p | -0.57 | 12.59 | 6.62 | 0.010 | 0.053 |
| mmu-miR-27b-3p | -0.56 | 13.55 | 4.99 | 0.025 | 0.094 |
| mmu-miR-101b-3p | -0.49 | 10.35 | 5.91 | 0.015 | 0.072 |
| mmu-miR-676-5p | -0.48 | 6.03 | 5.61 | 0.018 | 0.080 |
| mmu-miR-30a-5p | -0.48 | 14.34 | 5.41 | 0.020 | 0.083 |
| mmu-miR-126a-5p | -0.42 | 11.79 | 5.36 | 0.021 | 0.083 |
| mmu-miR-320-3p | 0.46 | 10.75 | 8.91 | 0.003 | 0.032 |
|  |  |  |  |  |  |
| **Up-regulated** |  |  |  |  |  |
| mmu-miR-138-5p | 0.51 | 5.96 | 5.62 | 0.018 | 0.080 |
| mmu-miR-28a-3p | 0.51 | 5.67 | 5.35 | 0.021 | 0.083 |
| mmu-miR-744-5p | 0.59 | 9.32 | 5.08 | 0.024 | 0.091 |
| mmu-let-7d-3p | 0.63 | 9.97 | 8.13 | 0.004 | 0.033 |
| mmu-miR-425-5p | 0.65 | 10.39 | 7.25 | 0.007 | 0.045 |
| mmu-miR-326-3p | 0.65 | 7.32 | 5.25 | 0.022 | 0.084 |
| mmu-miR-484 | 0.69 | 10.45 | 8.03 | 0.005 | 0.033 |
| mmu-miR-1198-5p | 0.72 | 9.51 | 7.65 | 0.006 | 0.039 |
| mmu-miR-151-5p | 0.73 | 7.42 | 7.51 | 0.006 | 0.040 |
| mmu-miR-151-3p | 0.74 | 11.94 | 5.73 | 0.017 | 0.079 |
| mmu-miR-181b-5p | 0.75 | 8.23 | 9.19 | 0.002 | 0.032 |
| mmu-miR-191-5p | 0.76 | 15.14 | 7.02 | 0.008 | 0.046 |
| mmu-miR-188-5p | 0.78 | 3.75 | 6.74 | 0.009 | 0.051 |
| mmu-miR-425-3p | 0.83 | 4.67 | 7.03 | 0.008 | 0.046 |
| mmu-miR-345-3p | 0.85 | 5.52 | 16.71 | 4.36E-05 | 0.003 |
| mmu-miR-1249-3p | 0.87 | 5.00 | 6.54 | 0.011 | 0.054 |
| mmu-miR-342-3p | 0.87 | 6.72 | 5.63 | 0.018 | 0.080 |
| mmu-miR-150-5p | 0.95 | 11.21 | 8.51 | 0.004 | 0.032 |
| mmu-miR-223-5p | 0.96 | 4.76 | 8.79 | 0.003 | 0.032 |
| mmu-miR-222-3p | 0.96 | 9.24 | 8.70 | 0.003 | 0.032 |
| mmu-miR-674-3p | 0.98 | 5.58 | 11.03 | 0.001 | 0.020 |
| mmu-miR-351-5p | 0.99 | 7.26 | 4.84 | 0.028 | 0.098 |
| mmu-miR-501-3p | 1.00 | 6.18 | 10.20 | 0.001 | 0.023 |
| mmu-miR-296-5p | 1.00 | 4.34 | 7.55 | 0.006 | 0.040 |
| mmu-miR-330-3p | 1.01 | 5.80 | 12.90 | 3.29E-04 | 0.012 |
| mmu-miR-5113 | 1.11 | 3.86 | 12.36 | 4.38E-04 | 0.013 |
| mmu-miR-361-3p | 1.11 | 6.32 | 10.60 | 0.001 | 0.023 |
| mmu-miR-183-5p | 1.17 | 5.54 | 5.31 | 0.021 | 0.083 |
| mmu-miR-1968-5p | 1.18 | 3.59 | 10.05 | 0.002 | 0.024 |
| mmu-miR-664-5p | 1.20 | 4.16 | 9.32 | 0.002 | 0.032 |
| mmu-miR-423-5p | 1.24 | 12.03 | 8.37 | 0.004 | 0.032 |
| mmu-miR-219a-1-3p | 1.25 | 3.16 | 5.46 | 0.020 | 0.083 |
| mmu-miR-328-3p | 1.26 | 11.63 | 8.56 | 0.003 | 0.032 |
| mmu-miR-423-3p | 1.28 | 11.00 | 8.64 | 0.003 | 0.032 |
| mmu-miR-205-5p | 1.30 | 8.06 | 18.66 | 1.56E-05 | 0.002 |
| mmu-miR-511-5p | 1.35 | 4.02 | 6.86 | 0.009 | 0.049 |
| mmu-miR-877-5p | 1.47 | 4.89 | 8.48 | 0.004 | 0.032 |
| mmu-miR-146a-5p | 1.57 | 8.36 | 8.17 | 0.004 | 0.033 |
| mmu-miR-1964-3p | 1.62 | 6.23 | 12.26 | 4.63E-04 | 0.013 |
| mmu-miR-3473d | 1.77 | 4.94 | 9.14 | 0.003 | 0.032 |
| mmu-miR-877-3p | 1.78 | 3.01 | 10.44 | 0.001 | 0.023 |
| mmu-miR-335-3p | 1.86 | 4.54 | 26.04 | 3.34E-07 | 9.91E-05 |
| mmu-miR-150-3p | 2.06 | 4.26 | 7.05 | 0.008 | 0.046 |
| mmu-miR-1983 | 2.54 | 2.67 | 13.70 | 2.15E-04 | 0.009 |
| mmu-miR-5107-5p | 2.68 | 3.69 | 7.81 | 0.005 | 0.037 |

**Suppl. Table 3** - Pathways regulated by target genes of miRNAs regulated by the genotype by age interaction in IGHD subjects

| **KEGG pathway** | **P-value** | **Genes** | **miRNAs** |
| --- | --- | --- | --- |
| Fatty acid biosynthesis | 7.62E-12 | 4 | 3 |
| Prion diseases | 3.04E-08 | 10 | 7 |
| Hippo signaling pathway | 3.18E-08 | 69 | 12 |
| Mucin type O-Glycan biosynthesis | 2.01E-07 | 13 | 8 |
| Signaling pathways regulating pluripotency of stem cells | 1.38E-06 | 64 | 12 |
| Glioma | 1.18E-05 | 32 | 13 |
| Proteoglycans in cancer | 1.37E-05 | 77 | 13 |
| Thyroid hormone signaling pathway | 1.63E-05 | 52 | 13 |
| Long-term depression | 3.17E-05 | 31 | 10 |
| Amphetamine addiction | 1.08E-04 | 32 | 9 |
| Prostate cancer | 1.10E-04 | 45 | 12 |
| ErbB signaling pathway | 1.62E-04 | 42 | 13 |
| Non-small cell lung cancer | 3.21E-04 | 29 | 11 |
| FoxO signaling pathway | 3.21E-04 | 61 | 12 |
| Prolactin signaling pathway | 3.21E-04 | 35 | 12 |
| Nicotine addiction | 4.05E-04 | 19 | 11 |
| Choline metabolism in cancer | 0.001 | 47 | 13 |
| Renal cell carcinoma | 0.001 | 33 | 11 |
| mTOR signaling pathway | 0.001 | 32 | 11 |
| TGF-beta signaling pathway | 0.001 | 37 | 10 |
| AMPK signaling pathway | 0.001 | 56 | 12 |
| Glutamatergic synapse | 0.001 | 48 | 11 |
| Dopaminergic synapse | 0.001 | 58 | 11 |
| Adrenergic signaling in cardiomyocytes | 0.001 | 51 | 11 |
| Endometrial cancer | 0.002 | 26 | 11 |
| MAPK signaling pathway | 0.002 | 99 | 12 |
| Type II diabetes mellitus | 0.002 | 25 | 11 |
| Ras signaling pathway | 0.002 | 89 | 12 |
| Sphingolipid signaling pathway | 0.002 | 51 | 12 |
| Focal adhesion | 0.002 | 84 | 12 |
| Circadian rhythm | 0.002 | 18 | 9 |
| Colorectal cancer | 0.003 | 28 | 11 |
| Rap1 signaling pathway | 0.003 | 82 | 12 |
| Melanoma | 0.004 | 33 | 12 |
| Insulin signaling pathway | 0.004 | 59 | 11 |
| Gap junction | 0.004 | 36 | 11 |
| Fatty acid metabolism | 0.004 | 14 | 7 |
| Ubiquitin mediated proteolysis | 0.005 | 58 | 11 |
| Long-term potentiation | 0.005 | 32 | 10 |
| Wnt signaling pathway | 0.005 | 56 | 10 |
| Pathways in cancer | 0.006 | 137 | 14 |
| Oxytocin signaling pathway | 0.006 | 62 | 12 |
| Transcriptional misregulation in cancer | 0.008 | 64 | 12 |
| Pancreatic cancer | 0.008 | 30 | 10 |
| mRNA surveillance pathway | 0.011 | 39 | 11 |
| Axon guidance | 0.011 | 51 | 11 |
| Cocaine addiction | 0.011 | 22 | 9 |
| Lysine degradation | 0.011 | 16 | 8 |
| Glycosaminoglycan biosynthesis | 0.011 | 10 | 6 |
| Neurotrophin signaling pathway | 0.012 | 50 | 13 |
| Phosphatidylinositol signaling system | 0.012 | 33 | 12 |
| Acute myeloid leukemia | 0.013 | 26 | 13 |
| PI3K-Akt signaling pathway | 0.015 | 119 | 12 |
| B cell receptor signaling pathway | 0.015 | 32 | 11 |
| Hedgehog signaling pathway | 0.015 | 24 | 9 |
| Dorso-ventral axis formation | 0.015 | 15 | 7 |
| GABAergic synapse | 0.022 | 33 | 10 |
| cGMP-PKG signaling pathway | 0.027 | 62 | 12 |
| Maturity onset diabetes of the young | 0.031 | 10 | 8 |
| Protein processing in endoplasmic reticulum | 0.036 | 62 | 10 |

**Suppl. Table 4** – Pathways regulated by target genes of miRNAs regulated by the genotype

| **KEGG pathways from up-regulated miRNAs** | **P-Value** | **Genes** | **miRNAs** |
| --- | --- | --- | --- |
| Fatty acid biosynthesis | 4.61E-14 | 4 | 2 |
| TGF-beta signaling pathway | 1.74E-09 | 36 | 8 |
| FoxO signaling pathway | 3.52E-05 | 55 | 8 |
| Glioma | 2.97E-08 | 33 | 8 |
| Prion diseases | 5.39E-08 | 6 | 5 |
| Proteoglycans in cancer | 1.03E-07 | 74 | 11 |
| Signaling pathways regulating pluripotency of stem cells | 3.36E-07 | 58 | 11 |
| Axon guidance | 1.28E-05 | 58 | 7 |
| Prostate cancer | 2.16E-05 | 43 | 9 |
| Fatty acid metabolism | 3.52E-05 | 14 | 7 |
| Acute myeloid leukemia | 7.05E-05 | 29 | 9 |
| Wnt signaling pathway | 3.26E-04 | 50 | 11 |
| Endocytosis | 3.26E-04 | 81 | 9 |
| mTOR signaling pathway | 3.26E-04 | 30 | 9 |
| Gap junction | 3.26E-04 | 33 | 9 |
| Neurotrophin signaling pathway | 3.26E-04 | 51 | 9 |
| Mucin type O-Glycan biosynthesis | 3.26E-04 | 12 | 6 |
| AMPK signaling pathway | 3.79E-04 | 51 | 10 |
| Pathways in cancer | 4.10E-04 | 132 | 12 |
| cGMP-PKG signaling pathway | 0.001 | 64 | 10 |
| Ubiquitin mediated proteolysis | 0.001 | 56 | 10 |
| Circadian rhythm | 0.001 | 18 | 7 |
| Adrenergic signaling in cardiomyocytes | 0.002 | 51 | 9 |
| ErbB signaling pathway | 0.002 | 36 | 9 |
| Long-term depression | 0.002 | 26 | 7 |
| Melanoma | 0.002 | 31 | 7 |
| Pancreatic cancer | 0.005 | 29 | 8 |
| Adherens junction | 0.006 | 33 | 9 |
| Regulation of actin cytoskeleton | 0.006 | 75 | 9 |
| Ras signaling pathway | 0.007 | 75 | 9 |
| Oocyte meiosis | 0.007 | 42 | 10 |
| Prolactin signaling pathway | 0.007 | 28 | 7 |
| Insulin signaling pathway | 0.010 | 52 | 11 |
| **KEGG pathways from down-regulated miRNAs** |  |  |  |
| Biotin metabolism | 3.41E-06 | 1 | 1 |
| Lysine degradation | 0.038 | 6 | 4 |
| Thyroid hormone signaling pathway | 0.040 | 12 | 5 |
